# Electrophysiological properties of layer 2/3 pyramidal neurons in the primary visual cortex of a retinitis pigmentosa mouse model (*rd10*)

**DOI:** 10.3389/fncel.2023.1258773

**Published:** 2023-09-15

**Authors:** Claas Halfmann, Thomas Rüland, Frank Müller, Kevin Jehasse, Björn M. Kampa

**Affiliations:** ^1^Systems Neurophysiology, Institute of Zoology, RWTH Aachen University, Aachen, Germany; ^2^Molecular and Cellular Physiology, Institute of Biological Information Processing (IBI-1), Forschungszentrum Jülich GmbH, Jülich, Germany; ^3^Research Training Group 2416 MultiSenses-MultiScales, RWTH Aachen University, Aachen, Germany; ^4^Research Training Group 2610 Innoretvision, RWTH Aachen University, Aachen, Germany; ^5^JARA BRAIN, Institute of Neuroscience and Medicine (INM-10), Forschungszentrum Jülich, Jülich, Germany

**Keywords:** visual cortex, *rd10* mouse model, retinitis pigmentosa, electrophysiology, patch-clamp

## Abstract

Retinal degeneration is one of the main causes of visual impairment and blindness. One group of retinal degenerative diseases, leading to the loss of photoreceptors, is collectively termed retinitis pigmentosa. In this group of diseases, the remaining retina is largely spared from initial cell death making retinal ganglion cells an interesting target for vision restoration methods. However, it is unknown how downstream brain areas, in particular the visual cortex, are affected by the progression of blindness. Visual deprivation studies have shown dramatic changes in the electrophysiological properties of visual cortex neurons, but changes on a cellular level in retinitis pigmentosa have not been investigated yet. Therefore, we used the *rd10* mouse model to perform patch-clamp recordings of pyramidal neurons in layer 2/3 of the primary visual cortex to screen for potential changes in electrophysiological properties resulting from retinal degeneration. Compared to wild-type C57BL/6 mice, we only found an increase in intrinsic excitability around the time point of maximal retinal degeneration. In addition, we saw an increase in the current amplitude of spontaneous putative inhibitory events after a longer progression of retinal degeneration. However, we did not observe a long-lasting shift in excitability after prolonged retinal degeneration. Together, our results provide evidence of an intact visual cortex with promising potential for future therapeutic strategies to restore vision.

## 1. Introduction

Retinitis pigmentosa (RP) is a group of retinal degenerative diseases responsible for the loss of photoreceptor cells, due to genetic mutations altering cellular signaling cascades (Hartong et al., 2006; Newton and Megaw, 2020). Yet retinal ganglion cells, which generate the output of the retina to the brain, remain intact for an extended time after disease onset. This initial resilience is rendering retinal ganglion cells a prime target for vision restoration methods. Several therapeutic approaches, such as optogenetic, pharmacological, or electrical stimulation, were used in animal models of retinitis pigmentosa to elicit activity in retinal and downstream circuits to restore vision or delay retinal degeneration (Aghaizu et al., 2017; Dias et al., 2018; Ayton et al., 2020; Farnum and Pelled, 2020; Kleinlogel et al., 2020; Prosseda et al., 2022). In human patients, electrical stimulation of retinal ganglion cells via retinal implants is able to elicit reliable responses in the visual cortex of RP patients, but the perception is usually restricted to light flashes or phosphenes (Humayun et al., 2012; Keserü et al., 2012; Yue et al., 2021; Wang et al., 2022). One reason for the limited success of these approaches, in addition to retinal changes, might be found in an altered visual system that has already undergone homeostatic changes due to the lack of visual input (Bhattacharyya, 2022; Caravaca-Rodriguez et al., 2022).

Primary visual cortex (V1) activity is susceptible to changes in sensory experience. Such changes are known to trigger modifications of cellular excitability by Hebbian as well as homeostatic plasticity mechanisms (Keck et al., 2017; Turrigiano, 2017). For example, a decrease in excitability is observed in layers 2 and 3 (L2/3) of pyramidal neurons (PNs) with development (Tatti et al., 2017; Ciganok-Hückels et al., 2023), together with an increase in functional inputs from the retina after eye-opening, leading to a refinement of the V1 network (Desai et al., 2002; Lu and Constantine-Paton, 2004; Ishikawa et al., 2014). In addition, visual deprivation experiments showed that the lack of correct visual input, especially around the critical period, leads to changes in rheobase, input resistance, and action potential threshold and shifts in the balance of excitatory and inhibitory inputs (Desai et al., 2002; Maffei and Turrigiano, 2008; Lambo and Turrigiano, 2013; Kannan et al., 2016). Some of these effects are not restricted to the critical period as the induction of homeostatic plasticity is also possible in adult animals (Goel and Lee, 2007; Wen and Turrigiano, 2021). Therefore, retinal activity is crucial to sustain the functional activity of V1. However, when the retina is subject to degeneration, it might also trigger neuronal homeostatic plasticity mechanisms, which could potentially counteract a successful vision restoration approach on a retinal level (Bhattacharyya, 2022; Caravaca-Rodriguez et al., 2022). Despite the great amount of literature on visual deprivation, remarkably little is known about the integrity of the visual cortex in retinitis pigmentosa. Few studies in murine retinitis pigmentosa models have shown a transient alteration in excitation and inhibition balance (Pietra et al., 2021), a reduction of dendritic spine density in L5 PNs (Martinez-Galan et al., 2022), and preserved synaptic plasticity (Begenisic et al., 2020). In addition, mouse models of other retinal degenerative diseases, such as glaucoma, also show adjustments in morphological and electrophysiological properties in areas downstream of the retina (Bhandari et al., 2019; Van Hook et al., 2021; Bhattacharyya, 2022). However, a detailed analysis of the electrophysiological properties of V1 neurons is still missing.

Therefore, we screened the electrophysiological properties of L2/3 PNs in V1 of the *rd10* mouse model of retinitis pigmentosa. In this model, the loss of rod photoreceptor cells starts after eye-opening around p18 and peaks around p30 (Chang et al., 2007; Gargini et al., 2007; Rösch et al., 2014). We used patch-clamp recording to compare the passive, active, and network properties of *rd10* and wild-type C57BL/6 mice. We recorded before and right after the critical period (Desai et al., 2002; Hooks and Chen, 2007; Espinosa and Stryker, 2012), and in *rd10* mice, this intersects with a maximum of degeneration of retinal outer nuclear layer (ONL) cells (Chang et al., 2007; Gargini et al., 2007; Rösch et al., 2014). We also included a later adult stage to test visual cortex physiology after prolonged retinal degeneration and impaired visual input. In juvenile *rd10* mice, after the critical period, we observed an increased intrinsic excitability profile within L2/3, but these changes were not permanent. In adult mice, after prolonged retinal degeneration, we only observed a slight increase in the current amplitude of spontaneous putative inhibitory events. Overall, the visual cortex of *rd10* mice remained similar to its wild-type counterpart, providing evidence for an intact visual cortex as required for future therapeutic strategies to restore vision.

## 2. Materials and methods

### 2.1. Animals

All mice were bred and housed according to standards and guidelines set by the European Directive on the Protection of Animals used for Scientific Purposes (2010/63/EU), the Federation of European Laboratory Animal Science Associations (FELASA), and the German Animal Welfare Act [Tierschutzgesetz (TierSchG), 2006]. Mice of both sexes were killed in accordance with the State Agency for Nature, Environment, and Consumer Protection (LANUV) of the State of North Rhine-Westphalia and supervised by the local animal welfare office (Institute for Laboratory Animal Science, RWTH Aachen). Two strains of mice were used: B6.CXB1-Pde6b^rd10^/J (*rd10*), from the local animal facility (Institute of Laboratory Animal Science, RWTH Aachen), and C57BL/6J (B6), initially from Charles River Laboratories, as a control group. *Rd10* mice harbor a mutation in the gene encoding the beta-subunit of rod cGMP phosphodiesterase-6 (Pde6b), leading to retinal degeneration (Chang et al., 2007; Chang, 2016). Mice were housed under a normal 12-h light/dark cycle with access to food and water *ad libitum*. Both strains did not receive any enrichment as this might interfere with the severity of retinal degeneration (Barone et al., 2014).

We used three age groups (I, II, and III) to monitor the electrophysiological properties of the visual cortex L2/3 PNs. As our first age group, we chose a time period shortly after eye-opening and before the onset of the critical period for visual experience-dependent development (Hooks and Chen, 2007; Espinosa and Stryker, 2012) as well as retinal degeneration in *rd10* mice (Chang et al., 2007) [age group I: pre-critical period p14–p16 (median age of mice in age group I B6: p14, *rd10*: p15.5; *n* = 3 and 4 mice, respectively)]. Since eye-opening occurs around p12 to p14 (Hoy and Niell, 2015; Cheng et al., 2022; Ciganok-Hückels et al., 2023), we only included animals that had fully opened eyes. The second age group covered a time period around the end of the critical period, in *rd10* mice this intersects with a maximum degeneration of retinal outer nuclear layer (ONL) cells (Chang et al., 2007; Gargini et al., 2007; Rösch et al., 2014) and the start of aberrant retinal activity (Stasheff et al., 2011; Biswas et al., 2014; Rösch et al., 2014) [age group II: post-critical period p29–p42 (median age of mice in age group II B6: p33, *n* = 21 mice; *rd10*: p31, *n* = 28 mice)]. The third age group consisted of a broader range of adult mice in which death of both rod and cone cells, as well as further retinal alterations in the form of oscillation and hyperactivity, occur in *rd10* mice (Chang et al., 2007; Gargini et al., 2007; Barhoum et al., 2008; Stasheff et al., 2011; Pennesi et al., 2012; Biswas et al., 2014; Rösch et al., 2014) [age group III: adult p59–p100 (median age of mice in age group III B6: p89, *n* = 29 mice; *rd10*: p90, *n* = 31 mice)]. For sub- and suprathreshold properties, we also included an additional supplementary age group before eye-opening [p10–p11 (median age of mice in age group pre-eye open B6: p11, *rd10*: p10.5, *n* = 2 mice for each genotype)]. In this group, we only included animals whose eyelids were completely closed. A detailed overview of the number of mice and recorded cells is provided as Supplementary Tables.

### 2.2. Acute brain slice preparation

Mice were anesthetized with isoflurane (AbbVie, UK) and killed by decapitation. The brain was quickly submerged in ice-cold slicing-artificial cerebrospinal fluid (aCSF) containing (in mM) 125 NaCl, 2.5 KCl, 1.25 NaH2PO4, 25 NaHCO3, 25 glucose, 6 MgCl2, 1 CaCl2, pH 7.4 (95% O2/5% CO2 and ~310 mOsm/l). Coronal slices (300 μm) were cut with a vibratome (VT1200S, Leica Biosystems, Germany) and then incubated at approximately 34°C for 40–60 min in regular aCSF containing (in mM): 125 NaCl, 2.5 KCl, 1.25 NaH2PO4, 25 NaHCO3, 25 Glucose, 1 MgCl2, 2 CaCl2, pH 7.4 (95% O2/5% CO2 and ~310 mOsm/l), before being stored at room temperature.

### 2.3. Electrophysiology

#### 2.3.1. Recording conditions

Slices were placed in a recording chamber (Luigs & Neumann, Germany) and continuously superfused with heated (32 ± 1°C) aCSF. The recording chamber was integrated into an upright microscope (LNscope, Luigs & Neumann, Germany). The primary visual cortex was identified by the visual inspection of slices with a 4x air objective (Olympus, Japan) and comparison of gray and white matter shape and borders with respect to reference brain atlantes (Paxinos, Allen Brain Institute). A 40x water immersion objective (Olympus, Japan) with infrared-Dodt (Luigs & Neumann, Germany) gradient contrast to a CMOS camera (Chameleon USB 3.0 monochrome camera, Point Gray-Teledyne FLIR, OR, USA) was used to visualize neurons. L2/3 was identified by distance to pia (100–300 μm), and PNs were selected based on the presence of an apical dendrite in combination with a soma shape. Patch pipettes (4–8 MΩ) were pulled from borosilicate filamented glass (GB150F-10, Scientific Products GmbH, Germany) with a horizontal pipette puller (P-1000, Sutter Instruments, Novato, CA, USA). For all experiments, pipettes were filled with an internal solution containing (in mM): 135 K-gluconate, 4 KCl, 10 HEPES, 4 Mg-ATP, 0.3 Na-GTP, 10 Na2-phosphocreatine, pH 7.2 adjusted with KOH (300 mOsm/l).

Whole-cell patch-clamp recordings were performed in voltage and current clamp with ELC-03XS and ELC-01MX amplifiers (NPI Electronic, Germany). Signals were low-pass filtered at 10 kHz and digitized at 20 kHz with a data acquisition board (PCle 6323, National Instruments, TX, USA) connected to a computer (Dell, Windows 8, 64-bit). Access resistance and electrode capacitance were compensated directly at the amplifier, while all other experimental settings were controlled by the recording software (WaveSurfer version 0.965, Howard Hughes Medical Institute, Janelia Research Campus, USA, https://wavesurfer.janelia.org/) running with MATLAB 2018 (MathWorks, CA, USA). Recorded data were not corrected for liquid junction potential. Data were not included in the analysis if resting membrane potential was unstable, visible as severe changes in membrane potential ~10 mV or more around the stimulation time, or if access resistance was higher than 40 MΩ.

#### 2.3.2. Sub- and suprathreshold recordings

Sub- and suprathreshold electrophysiological properties were recorded in aCSF by injecting 500 ms current steps from −100 to 300 pA for the age group I (and supplementary age group pre-eye-opening) and from −100 to 500 pA for age groups II and III. Current was injected in 50 pA increments, with an additional current step at −30 pA.

The stimulation pattern was recorded for 10 consecutive trials, properties were calculated on single sweeps and averaged across all 10 trials. Resting membrane potential was measured at 0 pA current step. Input resistance was obtained by the slope of the current–voltage relationship from −50 to 0 pA current steps. The time constant of the membrane was calculated with a mono-exponential fit to voltage changes resulting from a −50 pA current step. Voltage sag was calculated as the percentage difference between the initial voltage response and the sustained steady-state voltage response to a current injection of −100 and −50 pA. Afterward, the two responses were linearly fitted and interpolated to a response that would cause a hyperpolarization of −7.5 mV (van Aerde and Feldmeyer, 2015; Ciganok-Hückels et al., 2023).

Action potential (AP) properties were calculated from the first action potential. The AP threshold was calculated with the first derivative of the action potential, the threshold was set as the value where the mean of the first AP derivative exceeded two standard deviations. AP amplitude was calculated as the total voltage change from AP threshold to AP maximum. AP rise time was calculated as the time to reach maximal AP amplitude from the AP threshold. AP half-width was calculated as the full width at half-maximal AP amplitude. Rheobase was set as the first current step that was able to elicit action potentials. For a subset of cells, rheobase was subsequently checked with 10 pA current steps, oriented around the rheobase value that we found with the 50 pA current steps. Both mean values (rheobase with 50 pA steps and 10 pA steps) were very similar (data not shown); therefore, we combined both values. Area under curve (a.u.c.) values of frequency–current (FI) curves were calculated by trapezoidal numerical integration. For AP amount in 100 ms time bins of stimulation time, the APs in each respective bin were counted at fixed current steps (age group I 250 pA, age groups II and III 400 pA). The inter-spike interval (ISI) was calculated for neurons with at least 10 APs, regardless of the current step, across the stimulation period. ISIs were calculated for each inter-spike interval by dividing ISI-n = 1–8 by the ninth ISI (ISI-n/ISI-9).

#### 2.3.3. Extracellular stimulation

For extracellular stimulation, a second glass pipette (stimulation electrode filled with aCSF) connected to an isolated stimulator (DS2A, Digitimer Ltd, UK) was brought close to the slice surface and to the recorded neuron. Extracellular stimulation was controlled at the stimulation device and triggered by the recording software. After a trigger, cathodic square pulse stimuli (0.2 ms duration) were generated, and the resulting response of the stimulated network was measured in the recorded cell. Calculations were done on averaged traces from 40 sweeps in total.

Current-clamp (CC) recordings were done at resting membrane potential, with both electrodes (recording and stimulation) present in L2/3. The strength of the stimulation electrode was set to a value that would elicit a clear visible response but no detrimental effects. The collective potential change at the soma was measured, resulting in a subthreshold depolarizing event at resting membrane potential. For 20-Hz stimuli, each sweep contained five stimuli, while for 40-Hz stimulation, the stimuli alternated one sweep with one stimulus followed by a sweep containing two stimuli, resulting in 20 sweeps with 40-Hz stimulation.

Voltage clamp (VC) recordings followed initial recordings of cell properties in CC. Membrane potential was clamped at −45 and +10 mV. Calculation of equilibrium potentials and electrochemical driving forces showed that recordings at −45 mV would allow us to record putative excitatory postsynaptic currents (EPSCs) and putative inhibitory postsynaptic currents (IPSCs), since −45 mV is sufficiently distant from our chloride reversal potential of −92 mV. Ultimately, currents analyzed at −45 mV are confined to putative EPSCs, while putative IPSCs were recorded at +10 mV. The stimulation was set up as described above, but here the recording electrode is in L2/3, while the stimulation electrode was placed perpendicularly below the recording electrode in L4. Stimulation strength was slowly raised until a clear, but not saturated, response was visible in the recording electrode. Calculations were done on averaged traces from 40 sweeps in total. Failures were excluded to better monitor the potential effect of retinal degeneration on event parameters.

#### 2.3.4. Spontaneous activity

For CC recordings, the membrane potential was raised to −50 and −40 mV by short (500 ms) test current injections. Once a current value to raise the membrane potential to the goal potential was determined, the test pulse was changed to the stimulation pulse of 120 s. Cells that did not show a strong divergence from the goal potential were used for analysis. During the stimulation, cells were able to fire action potentials or display subthreshold synaptic events that depolarized or hyperpolarized the membrane. For CC recordings of action potential frequency, we used only cells at −40 mV that displayed action potentials. Subthreshold events are putative excitatory or inhibitory postsynaptic potential (EPSP/IPSP). Since we did not use synaptic blockers, we referred to these events as depolarizing or hyperpolarizing events.

Depolarizing and hyperpolarizing events at −50 mV were detected on filtered (10 ms moving average) sweeps excluding action potentials by a MATLAB implementation of a detection algorithm by Clements and Bekkers (1997). For all event types and cells, the same template was used. The detection criterion was set to 4 × SD. In subsequent analysis, only events that were greater than 3 × SD of the membrane potential and had a rise time below 10 ms were accepted. These events were used to calculate amplitude and frequency.

Spontaneous events in VC were recorded at the respective clamped membrane potential (−45 mV, +10 mV). The same post-processing steps as with CC events were applied to VC events using the same detection algorithm (Clements and Bekkers, 1997). Since we did not use synaptic blockers, we referred to these events as putative EPSCs and putative IPSCs. For all event types, cells, and holding voltage steps, the same template was used, and the detection criterion was set to 5 × SD. Up to two 120-s sessions for CC and up to three for VC were recorded, inter-session interval of <10 s. Sessions values were averaged.

### 2.4. Data analysis and statistics

Data and statistical analysis were performed in MATLAB (MathWorks, versions 2019b and 2022a). Recorded data were accessed via MATLAB scripts provided by the recording software (WaveSurfer version 1.0.2, Howard Hughes Medical Institute, Janelia Research Campus, USA) and subsequently analyzed with custom-made scripts. Experiments and data analysis were not blinded to genotype.

For the statistical analysis, we performed non-parametric tests for our entire dataset, since the data were not normally distributed. For intra-age group differences (B6 vs. *rd10*), we used the independent, two-sided Wilcoxon rank-sum test. In addition to intra-age group differences, which are the main focus of our study, we also tested the respective inter-age group differences for sub- and suprathreshold electrophysiological properties of B6 and *rd10* (Kruskal–Wallis test with *post-hoc* Bonferroni corrected multiple comparisons). Our data followed a similar developmental trend described in previous studies for rodent primary visual cortex (Supplementary Tables 12–15) (Etherington and Williams, 2011; Tatti et al., 2017; Ciganok-Hückels et al., 2023), despite small differences in the change in rheobase current with age compared to the previous literature, which might be due to differences in internal solutions and age group distribution.

Data in figures and Supplementary Tables are presented as mean ± one standard deviation (SD). Significance levels are given as ^*^p < 0.05, ^**^p < 0.01, ^***^p < 0.001, and NS not significant (*p* > 0.05) and are displayed in the figures and Supplementary Tables. Sample sizes of recorded cells are presented as part of each figure caption. In addition, Supplementary Tables present a detailed overview of the number of mice and recorded cells.

## 3. Results

### 3.1. Gradual retinal degeneration does not lead to a long-lasting shift of sub- and suprathreshold membrane properties

**Figure 1:**
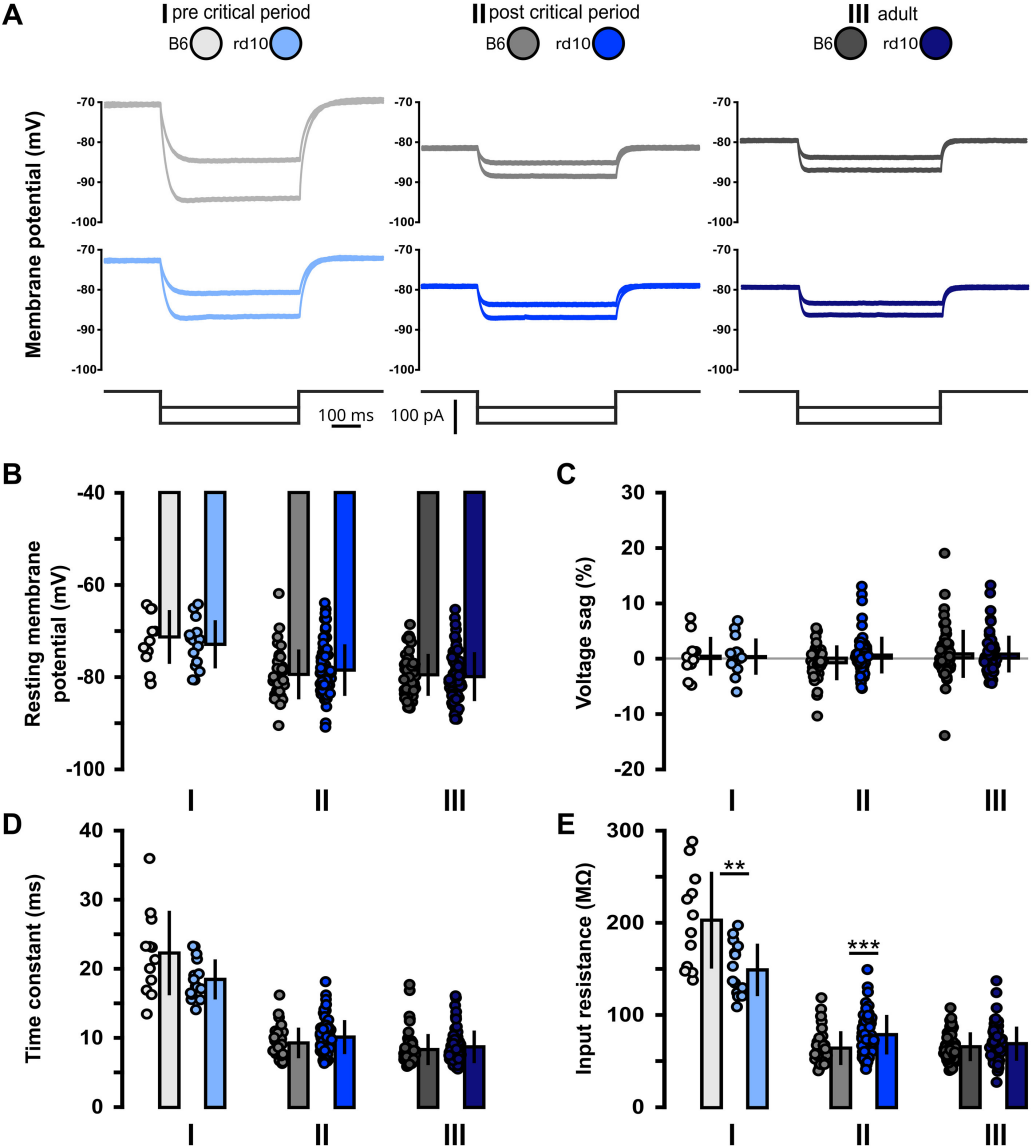
Acute but non-long-lasting shift of subthreshold membrane properties of *rd10* L2/3 PNs. **(A)** Examples of voltage responses to hyperpolarizing current steps for each of the three age groups (I, II, and III) and genotypes (gray traces B6, blue traces *rd10*). Bottom traces show injected current steps (−50 and −100 pA). **(B)** No change in resting membrane potential between B6 and *rd10* in all age groups. **(C)** Voltage sag is not changed between B6 and *rd10* in all age groups. **(D)** Time constant is not different between B6 and *rd10* in all age groups. **(E)** Input resistance of age groups I and II showed a significant difference between B6 and *rd10*. Sample size of recorded cells for **(B–E)** I: *n* = 12 B6; 15 *rd10*, II: *n* = 35 B6; 65 *rd10*, III: *n* = 60 B6; 66 *rd10*.

First, we recorded subthreshold properties to monitor changes in intrinsic excitability between B6 and *rd10* mice from each respective age group (Figure 1; Supplementary Table 1; age group I: p14–p16, II: p29–p42, III: p59–p100). In addition to these age groups, sub- and suprathreshold properties were tested in a supplementary age group before eye-opening, in this age group, *rd10* and B6 neurons showed no significant differences (Supplementary Figure 1; Supplementary Table 11). We found no significant difference in resting membrane potential (Figure 1B), voltage sag (Figure 1C), and membrane time constant (Figure 1D). However, the input resistance of age group I is significantly higher for B6 cells compared to *rd10* (Figure 1E, I: B6: 202.4 ± 52.3 MΩ, *rd10*: 148.7 ± 28.3 MΩ, ^**^p < 0.01, Wilcoxon rank-sum test). Moreover, *rd10* PNs from age group II had a significantly higher input resistance (Figure 1E, II: B6: 64.3 ± 18.3 MΩ, *rd10*: 78.8 ± 21.4 MΩ, ^***^p < 0.001, Wilcoxon rank-sum test). Within age group III, there was no significant difference. These results indicate that retinal degeneration has a small and short-lasting impact on the intrinsic excitability of L2/3 PNs, with a decreased input resistance right after eye-opening and before the onset of retinal degeneration and an increased input resistance after the critical period and progression of retinal degeneration.

**Figure 2:**
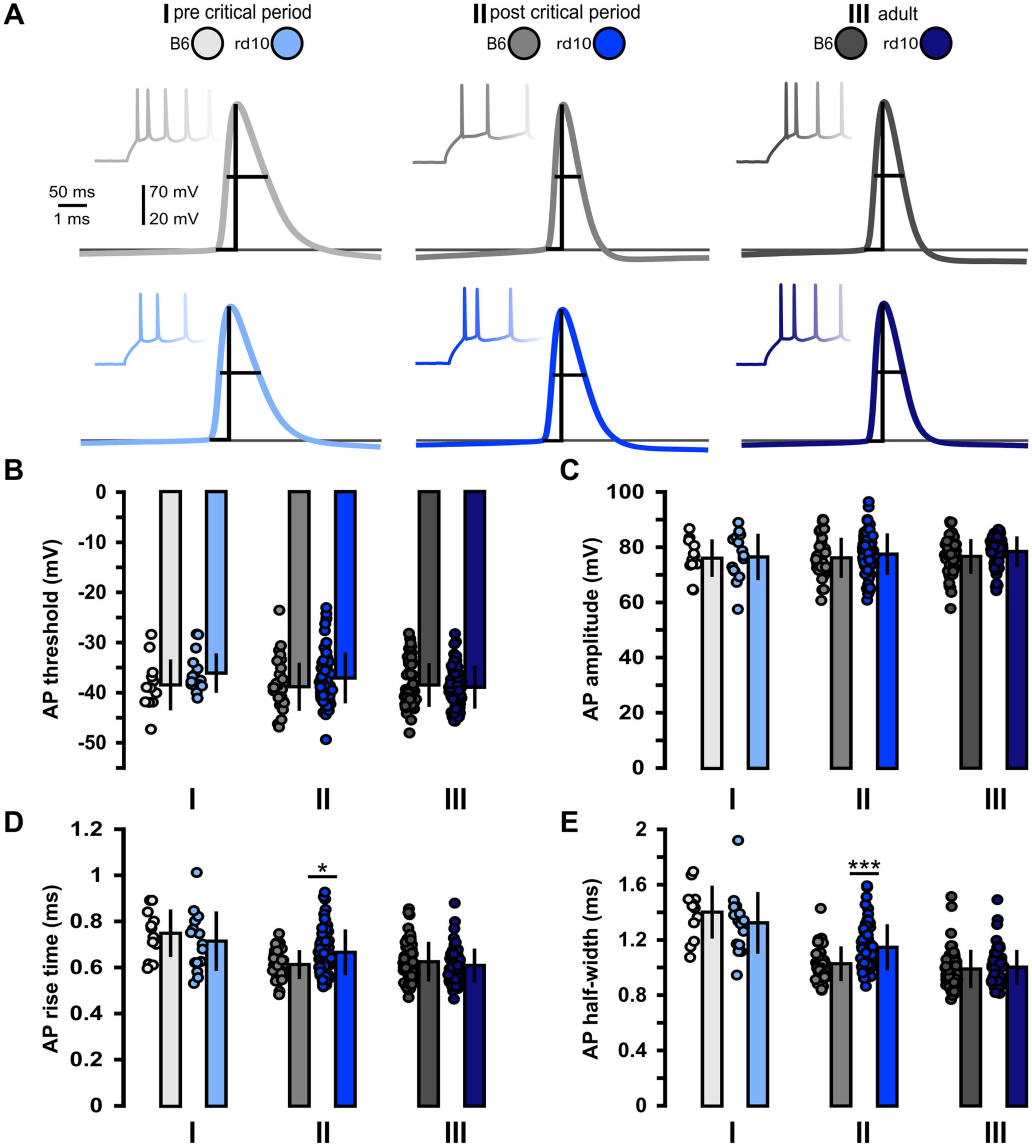
Acute but non-long-lasting changes in action potential properties in *rd10* L2/3 PNs. **(A)** Example traces of action potential firing elicited by depolarizing current steps. Traces in the background show the first few action potentials elicited by current step injections. Traces in front are examples of the first action potential of these trains for each of the three age groups (I, II, and III) and genotypes (gray traces B6, blue traces *rd10*). Vertical and horizontal lines indicate action potential amplitude and half-width, respectively, bottom line indicates action potential threshold. **(B)** Action potential threshold and **(C)** action potential amplitude did not show significant differences between B6 and *rd10*. **(D)** Action potential rise time and **(E)** Action potential half-width were significantly increased in *rd10* from age group II. Sample size of recorded cells for **(B–E)** I: *n* = 12 B6; 15 *rd10*, II: *n* = 35 B6; 65 *rd10*, III: *n* = 60 B6; 66 *rd10*.

Next, we looked at the suprathreshold properties to characterize the effect of retinal degeneration on the input–output properties of L2/3 PNs (Figure 2; Supplementary Table 1). Within all age groups, we found no significant differences between B6 and *rd10* AP threshold and amplitude (Figures 2B, C). In age group II, AP rise time was slightly increased in *rd10* L2/3 PNs compared to control animals (Figure 2D, age group II: B6: 0.61 ± 0.06 ms, *rd10*: 0.66 ± 0.09 ms, ^*^p < 0.05, Wilcoxon rank-sum test). Similarly, the AP half-width of *rd10* L2/3 PNs was slightly broader in comparison to B6 (Figure 2E, age group II: B6: 1.03 ± 0.12 ms, *rd10*: 1.15 ± 0.17 ms, ^***^p < 0.001, Wilcoxon rank-sum test). In age groups I and III, these two parameters were not significantly different from the control.

**Figure 3:**
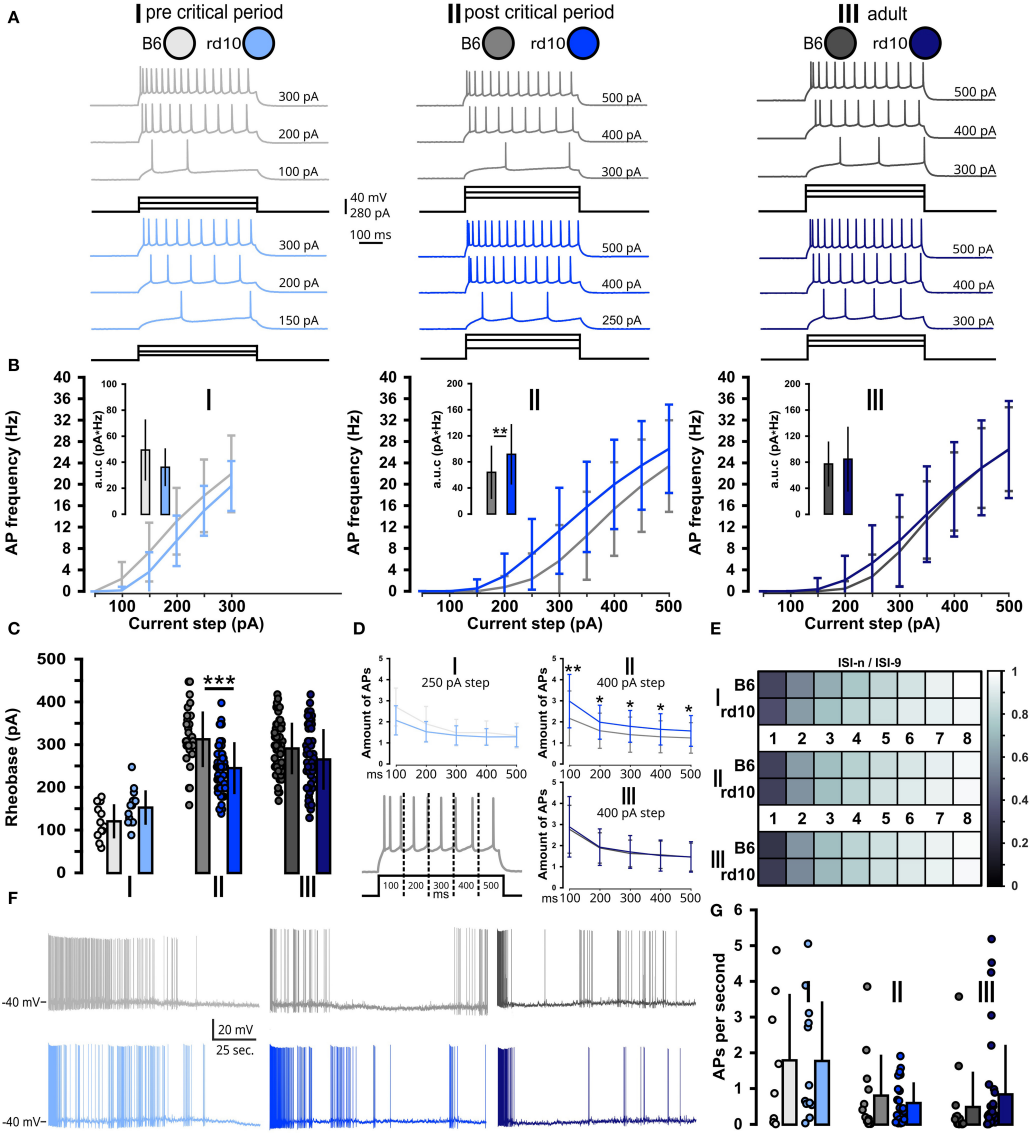
Acute but non-long-lasting shift of suprathreshold properties in *rd10* L2/3 PNs. **(A)** Example traces of action potential firing. For each of the three age groups (I, II, and III) and two genotypes (B6 gray traces and *rd10* blue traces), one exemplary cell is presented. Each of these cells is shown at three different depolarizing current steps, from rheobase to the maximal current step. **(B)** AP firing frequency at each depolarizing current step (FI curve). Insets show the area under the FI curve. Only *rd10* neurons from age group II had significantly higher evoked AP firing rates. **(C)** Rheobase of *rd10* L2/3 PNs from age group II is significantly decreased. **(D)** AP amount in 100 ms time bins of the stimulation period, dashed lines indicate the different time bins for analysis of AP firing in the respective time bin. Age group II had a significantly higher number of APs in each time bin. **(E)** Intensity plot of the adaptation ratio (ISI-n/ISI-9) did not reveal any differences between *rd10* and B6. **(F)** Example traces of ongoing AP firing while membrane potential is raised to −40 mV. **(G)** Quantification of spontaneous AP firing did not reveal significant differences between B6 and *rd10* across all age groups. Sample size of recorded cells for **(B–D)** I: *n* = 12 B6; 15 *rd10*, II: *n* = 35 B6; 65 *rd10*, III: *n* = 60 B6; 66 *rd10*, **(E)** I: *n* = 11 B6; 15 *rd10*, II: *n* = 22 B6; 48 *rd10*, III: *n* = 47 B6; 54 *rd10*, and **(G)** I: *n* = 8 B6; 12 *rd10*, II: *n* = 12 B6; 23 *rd10*, III: *n* = 14 B6; 33 *rd10*.

As we observed a slight increase in the excitability of L2/3 PNs after the onset of retinal degeneration in *rd10* mice, we also tested how this observation transfers to action potential firing by injecting increasing depolarizing currents steps (Figure 3A). Indeed, the resulting frequency–current (FI) curves showed an increase in evoked firing frequency for *rd10* mice in age group II compared to control (Figure 3B; Supplementary Table 2). The FI curves of the other age groups I and III were similar between the two mouse lines. To better quantify the increase in AP firing, we analyzed the mean area under curve (a.u.c.) values for B6 and *rd10* FI curves. For age groups I and III, we found no significant differences. For age group II, we observed a significantly greater area under the FI curve in *rd10* mice compared to B6, indicating increased action potential firing in *rd10* at age group II after the critical period (Figure 3B inset; age group II: B6 = 64.31 ± 41.14 pA^*^Hz, *rd10*: 92.22 ± 46.72 pA^*^Hz, ^**^p < 0.01, Wilcoxon rank-sum test; Supplementary Table 5). Similarly, the rheobase was smaller in *rd10* mice of age group II compared to control, also indicating an increased excitability of visual cortex neurons in the critical period (Figure 3C; age group II: B6: 314.3 ± 65.5 pA, *rd10*: 246.5 ± 61.1 pA, ^***^p < 0.001, Wilcoxon rank-sum test). No difference could be observed between the two mouse lines in age groups I and III (Supplementary Table 5). Furthermore, testing the AP amount in 100 ms time bins of the stimulation period revealed a significant increase in each time bin in APs for *rd10* in AG-II (time bin 1, ^**^p < 0.01, time bin 2–5, ^*^p < 0.05), age groups I and III did not show such a difference (Figure 3D; Supplementary Table 3), while for the AP adaptation ratio, we did not find any difference in any age group (Figure 3E; Supplementary Table 4).

In addition to evoked AP firing (Figures 3A–E), visual deprivation has been shown to also influence spontaneous AP firing in L4 and L2/3 (Maffei et al., 2004; Maffei and Turrigiano, 2008). For this, we brought the recorded neuron closer to the AP firing threshold by raising the membrane potential to −40 mV where small fluctuations evoked by spontaneous synaptic input from the surrounding network of L2/3 PNs or intrinsic properties can lead to AP induction (Figure 3F). Overall PNs presented a broad range of mean action potential firing, ranging from a few cells with higher spiking rate (2 Hz) to predominantly cells with lower spontaneous AP firing (below 1 Hz). Yet, no significant difference between B6 and *rd10* was found in any age group (Figure 3G; Supplementary Table 5).

Taken together, these results showed that in addition to the increased subthreshold excitability, *rd10* L2/3 PNs also became more likely to fire APs after the onset of retinal degeneration but only at the end of the critical period. B6 and *rd10* neurons expressed similar sub- and suprathreshold properties after full maturation of the visual system.

### 3.2. Gradual retinal degeneration does not lead to a long-lasting shift of pre- and postsynaptic network properties

**Figure 4:**
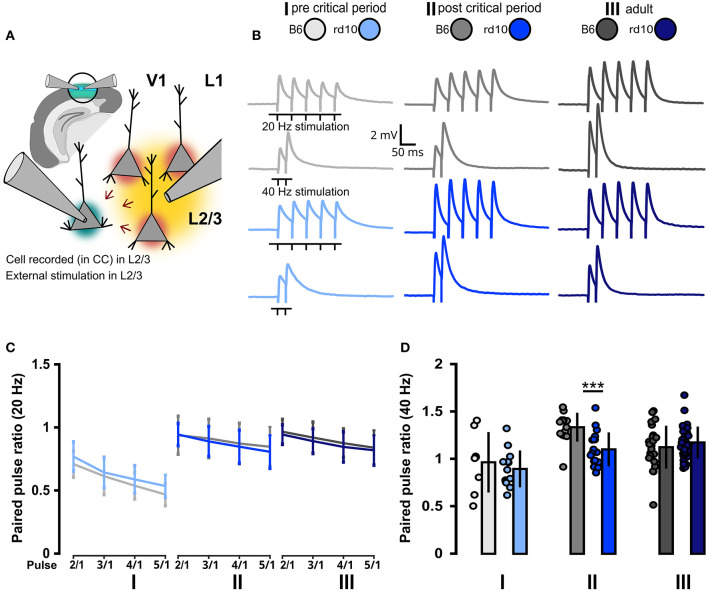
Consistent pre- and postsynaptic network properties in *rd10* and B6 L2/3 PNs. **(A)** Schematic representation of the experimental setup. V1 in the brain slice is highlighted in green. Layer 2/3 PNs were recorded in current-clamp mode (green highlighted cell), while a second pipette is used for the extracellular stimulation of the surrounding L2/3 neurons (yellow area of stimulation and red highlighted cells affected by stimulation). **(B)** Example traces of voltage responses evoked by the extracellular stimulation of the surrounding network (five stimuli at 20 Hz and two stimuli at 40 Hz). **(C)** Paired pulse ratios {PPR [event(n)/event(1)]} at 20 Hz did not reveal any difference between B6 and *rd10*. **(D)** Paired pulse ratios {PPR [event(2)/event(1)]} at 40 Hz show a significant difference between B6 and *rd10* in age group II. Sample size of recorded cells for **(C)** I: *n* = 8 B6; 13 *rd10*, II: *n* = 13 B6; 16 *rd10*, III: *n* = 21 B6; 29 *rd10*, **(D)** I: *n* = 8 B6; 13 *rd10*, II: *n* = 14 B6; 19 *rd10*, III: *n* = 24 B6; 37 *rd10*.

Visual deprivation leads not only to increased excitability of cortical neurons but also to changes in synaptic efficacy. Therefore, we investigated whether retinal degeneration also has an impact on synaptic short-term plasticity in the visual cortex. To address this, we recorded postsynaptic activity in L2/3 PNs by stimulating the surrounding neurons with an extracellular stimulation electrode placed in the same L2/3 in cortical brain slices of *rd10* or B6 mice (Figure 4A). The stimulation protocol consisted of trains of five stimuli at 20 Hz or two stimuli at 40 Hz (Figure 4B). We quantified the change in the postsynaptic response within these trains by calculating paired-pulse ratios (PPRs) between the response to the first stimulus and those to each of the following stimuli. PPRs for stimuli at 20 Hz were similar for *rd10* and B6 across all age groups (Figure 4C; Supplementary Table 6). However, at 40 Hz, the second pulse in a train was less efficient in *rd10* compared to B6 mice in age group II (Figure 4D, II: B6 = 1.33 ± 0.15, *rd10* = 1.10 ± 0.18, ^***^p < 0.001, Wilcoxon rank-sum test). This suggests an increased release probability in the presynaptic boutons of *rd10* neurons after the progression of retinal degeneration at the end of the critical period.

**Figure 5:**
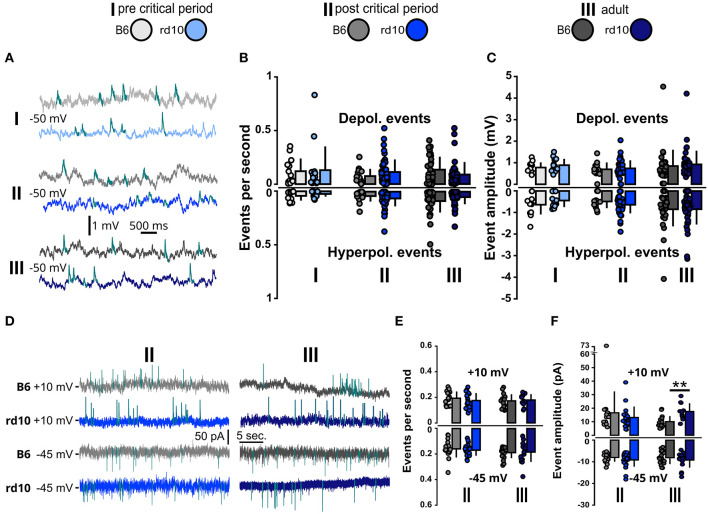
Spontaneous synaptic inputs are stable in *rd10* L2/3 PNs. **(A)** Example traces of spontaneous depolarizing events in the current clamp at membrane potential raised to −50 mV (for hyperpolarizing events, see Supplementary Figure 2). **(B)** Mean events per second for depolarizing and hyperpolarizing events measured at −50 mV membrane potential and **(C)** Mean median amplitudes of depolarizing and hyperpolarizing events remain similar between B6 and *rd10* across all age groups. **(D)** Example traces of spontaneous inhibitory and excitatory currents in voltage clamp at +10 mV (two upper sweeps) and −45 mV (two lower sweeps), respectively. **(E)** Mean events per second of spontaneous postsynaptic currents at +10 mV and −45 mV were not different between B6 and *rd10*. **(F)** Mean median amplitudes of spontaneous membrane currents at +10 mV and −45 mV. Age group III currents at +10 mV show a significant difference between B6 and *rd10*. Sample size of recorded cells for **(B, C)** I: *n* = 13 B6; 15 *rd10*, II: *n* = 14 B6; 44 *rd10*, III: *n* = 31 B6; 41 *rd10* cells, **(E, F)** (+10 mV): II: *n* = 16 B6; 19 *rd10*, III: *n* = 18 B6; 11 *rd10* and **(E, F)** (−45 mV): II: *n* = 18 B6; 24 *rd10*, III: *n* = 19 B6; 17 *rd10*.

We, therefore, also measured the occurrences of spontaneous excitatory and inhibitory postsynaptic potentials (Barnes et al., 2015; Alejandre-García et al., 2022) by raising the resting membrane potential to −50 mV (Figure 5A). The raised membrane potential increases the distance to the inhibitory reversal potential and therefore also allows simultaneous measurements of inhibitory postsynaptic potentials (see Supplementary Figure 2) (Sanchez-Vives and McCormick, 2000; Okun and Lampl, 2008). For spontaneous synaptic inputs in CC, we did not observe any difference between *rd10* and B6 mice, neither in event frequency (Figure 5B) nor in event amplitudes (Figure 5C; see also Supplementary Table 7). Next, we recorded spontaneous synaptic inputs in VC to better distinguish excitatory (−45 mV) and inhibitory (+10 mV) events (Figure 5D). Since retinal degeneration starts occurring in age group II, we only recorded this group and age group III to monitor a possible long-lasting change. We did not see significant changes neither in the frequency of synaptic events (Figure 5E; Supplementary Table 8) nor in inter-event interval (IEI) distribution (Supplementary Figure 3). However, we did observe a slightly increased amplitude of inhibitory currents for *rd10* mice in age group III (Figure 5F, age group III: B6: 10.05 ± 4.09 pA, *rd10* = 17.49 ± 6.15 pA, ^**^p < 0.01, Wilcoxon rank-sum test).

**Figure 6:**
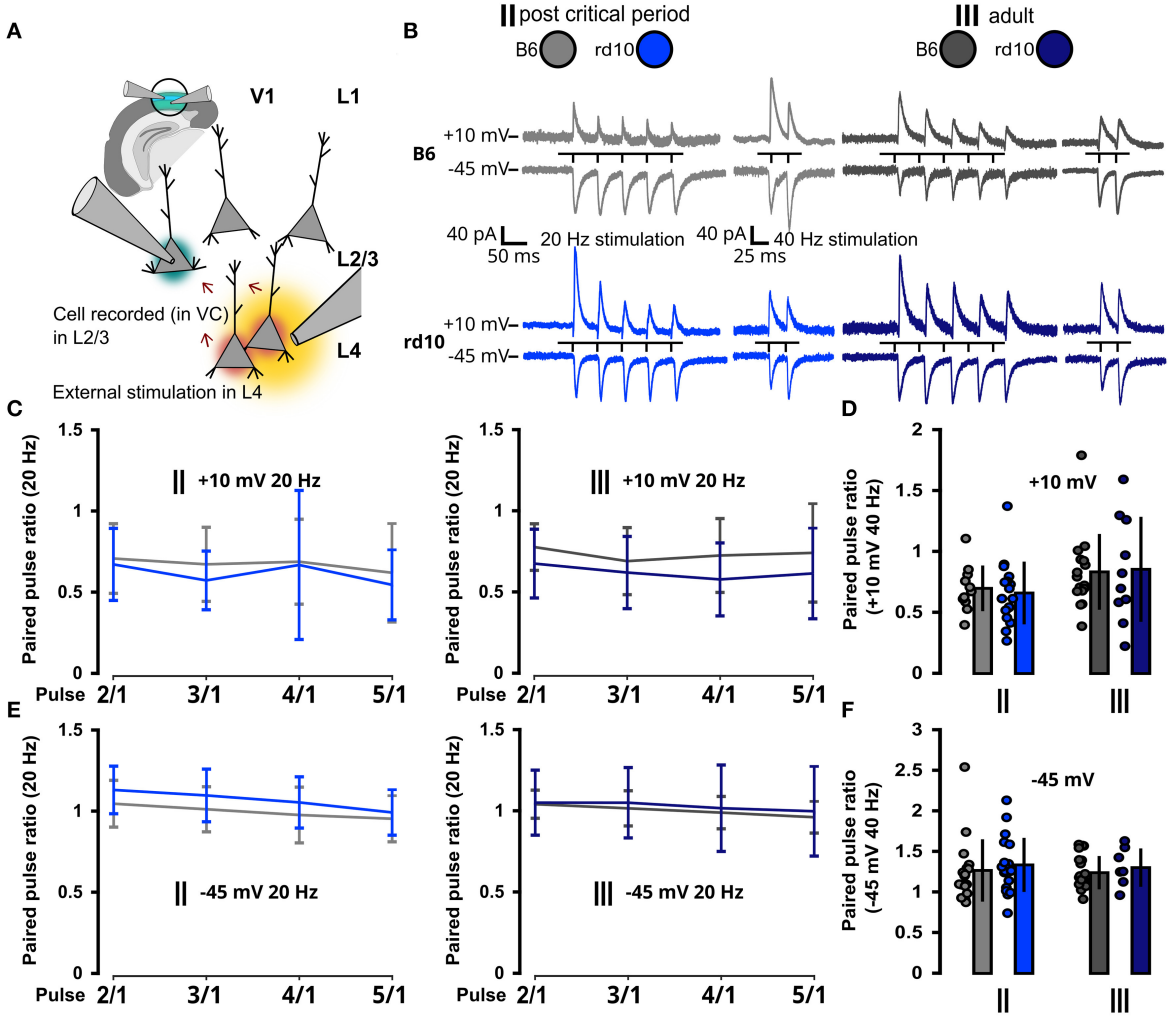
No change in synaptic properties from L4 in *rd10* L2/3 PNs. **(A)** Schematic representation of the experimental setup. V1 in the brain slice is highlighted in green. L2/3 PNs were recorded in voltage clamp mode (green highlighted cell), while a second pipette was used for the extracellular stimulation of L4 neurons (yellow area of stimulation and red highlighted cells affected by stimulation). **(B)** Example traces of postsynaptic current responses to stimulation in L4 recorded in L2/3 PNs at different holding membrane potentials (stimulation protocol was five stimuli at 20 Hz and two stimuli at 40 Hz). **(C)** Paired pulse ratio {PPR [event(n)/event(1)]} with 5 stimuli at 20 Hz at +10 mV. **(D)** Paired pulse ratio with two stimuli at 40 Hz at +10 mV. **(E)** Paired pulse ratio with five stimuli at 20 Hz at −45 mV. **(F)** Paired pulse ratio with two stimuli at 40 Hz at −45 mV. None of the paired pulse ratio values **(C–F)** was significantly different between B6 and *rd10*. Sample size of recorded cells for **(C)** II: *n* = 10 B6; 13 *rd10*, III: *n* = 12 B6; 8 *rd10*, **(D)** II: *n* = 11 B6; 17 *rd10*, III: *n* = 16 B6; 10 *rd10*, **(E)** II: *n* = 17 B6; 14 *rd10*, III: *n* = 19 B6; 7 *rd10*, **(F)** II: *n* = 18 B6; 20 *rd10*, III: *n* = 19 B6; 7 *rd10*.

Since visual input to the cortex is mainly transmitted from thalamocortical projections to L4 and from there further to L2/3, we also wanted to test whether synaptic short-term plasticity is altered between these layers after retinal degeneration. Therefore, we stimulated L4 and measured postsynaptic responses in L2/3 (Figures 6A, B). We recorded PPR in VC to better distinguish excitatory (−45 mV) and inhibitory (+10 mV) inputs. While changes in PPR could be a potential effect of retinal degeneration, we did not detect differences in PPR at 20 and 40 Hz for excitatory and inhibitory inputs from L4 neurons (Figures 6C–F; Supplementary Tables 9–10).

Taken together, these data suggest that direct inter-layer feedforward visual inputs to L2/3 PNs from L4 are not affected by retinal degeneration. Rather the L2/3 intra-layer network shows effects of retinal degeneration around the onset of the pathology, due to intrinsic changes in the excitability of L2/3 PNs.

## 4. Discussion

We performed an *in vitro* screening of electrophysiological properties in the *rd10* retinitis pigmentosa mouse model to test whether retinal degeneration affects L2/3 pyramidal neurons in V1. After the onset of retinal degeneration, the intrinsic excitability increased, but these changes were not long-lasting, since we observed no difference between B6 and *rd10* L2/3 PNs in adult mice with further progressive retinal degeneration. Moreover, we did not reveal long-lasting changes in synaptic efficacy in adult *rd10*. Only the current amplitude of spontaneous putative inhibitory events showed a slight increase after prolonged retinal degeneration. L2/3 cells in V1 of *rd10* mice did not develop an electrophysiological profile that was severely different from its wild-type counterpart.

While no severe changes exist in the visual cortex of *rd10* mice after retinal degeneration, our data show changes in several sub- and suprathreshold membrane properties (Figures 1, 2), an increase in excitability (Figure 3) and an increase in release probability for excitatory synaptic inputs (Figure 4) around the peak of retinal degeneration (age group II) (Chang et al., 2007; Gargini et al., 2007; Rösch et al., 2014). This increase in the excitability of L2/3 PNs is similar to what has been observed after prolonged visual deprivation (Maffei and Turrigiano, 2008; Lambo and Turrigiano, 2013). Furthermore, prolonged exposure of adult mice to darkness for up to 5 weeks triggered an increase in the resting membrane potential and input resistance as well as a decrease in the rheobase current, together making the L2/3 PNs in V1 more sensitive to synaptic input (Brown et al., 2019). In addition to changes in intrinsic excitability, we observed a decreased PPR within L2/3 in *rd10* age group II at the end of the critical period, suggesting an increased release probability, which rebounds back to control levels after prolonged retinal degeneration in age group III. It has been shown previously that changes in activity and sensory input can lead to the accumulation of synaptic vesicles (Nahmani and Turrigiano, 2014), which could be connected to an increase in readily releasable pool size (Murthy et al., 2001; Han and Stevens, 2009). In addition, activity changes are known to influence release probability (Maffei and Turrigiano, 2008; Zhao et al., 2011; Kannan et al., 2016; Zhuang et al., 2020). Retinal degeneration is also known to alter the release probability of retinal ganglion cells (Bhandari et al., 2019), while for *rd10* an increase in evoked release of cortical neurotransmitters, which rebounds back to baseline in older animals, has been described (Pietra et al., 2021). These results, combined with our data shown here, highlight that gradual retinal degeneration in *rd10* mice shows similarities to the effects of visual deprivation studies.

In age group III, we saw an increase in the amplitude of spontaneous putative IPSCs (Figure 5). Changes in visual input around the critical period are well-known to affect inhibitory properties in L4 (Maffei et al., 2006, 2010; Nahmani and Turrigiano, 2014) and L2/3 (Gao et al., 2014, 2017; Kannan et al., 2016). A change in mIPSC frequency (Kannan et al., 2016; Gao et al., 2017) and a shift of the excitation to inhibition ratio toward inhibition (Kannan et al., 2016), in combination with an upregulation of excitatory components (Hengen et al., 2013; Lambo and Turrigiano, 2013), are thought to counteract the loss of visual input and reestablish a firing rate set point (Turrigiano, 2017). For *rd10* in age group III, the result of such homeostatic processes seems to be an increase in inhibitory amplitude. Interestingly, our data are similar to those from visual deprivation experiments when patterned vision is lost for both eyes via binocular lid sutures. In this case, the effects are often less pronounced than those with monocular lid suture, as shown in *in vivo* and *in vitro* experiments (Gordon and Stryker, 1996; Sato and Stryker, 2008; Lambo and Turrigiano, 2013). Together, it can be suggested that Hebbian and homeostatic plasticity effects also occur after gradual retinal degeneration, leading to a temporary shift in electrophysiological properties at the peak of retinal degeneration.

In addition to retinal degeneration in *rd10* mice, normal development and aging also influence electrophysiological properties. Studies that follow maturing cells in the cortex report age-dependent changes in morphological and electrophysiological properties. These changes are well-documented for several brain areas (Zhang, 2004; Oswald and Reyes, 2008; Kroon et al., 2019; Perez-García et al., 2021), including rodent primary visual cortex (Kasper et al., 1994; Etherington and Williams, 2011; Tatti et al., 2017; Ciganok-Hückels et al., 2023). Our overall wild-type B6 and *rd10* sub- and suprathreshold data (Figures 1–3) are in accordance with the time course and extent of developmental electrophysiological changes presented in these studies. Furthermore, we show that neurons in V1 of *rd10* mice are similar to control at an age before eye-opening and before the onset of retinal degeneration (Supplementary Figure 1; Supplementary Table 11). A diversity of molecular mechanisms are responsible for such changes in intrinsic excitability, including different types of ion channels (Wefelmeyer et al., 2016; Debanne and Russier, 2019; Debanne et al., 2019). Depriving cultured pyramidal neurons of activity increases their excitability by reducing the persistent potassium current and increasing voltage-gated sodium currents (Desai et al., 1999). Whereas, in L5 PNs, visual deprivation reduces excitability by increasing a persistent voltage-gated potassium current (Nataraj et al., 2010). These mechanisms are likely to be also involved in age- or development-induced changes of intrinsic excitability in the visual cortex (Etherington and Williams, 2011; Tatti et al., 2017; Ciganok-Hückels et al., 2023) and similarly in the observed changes in *rd10* mice.

While retinal degeneration leads to the loss of photoreceptors, retinal ganglion cells remain intact and can still produce a retinal output via intrinsic activity or upon reactivation, which is projected downstream to central brain regions including V1 (Mazzoni et al., 2008; Ivanova et al., 2016; Sahel et al., 2021; Yue et al., 2021). Ongoing ganglion cell activity could, therefore, provide input to V1 even without visual input or functional photoreceptors. Indeed, because of photoreceptor loss, the remaining retina develops aberrant oscillatory activity in mouse models of retinitis pigmentosa (*rd1* and *rd10*) (Margolis et al., 2008; Biswas et al., 2014; Goo et al., 2016; Ivanova et al., 2016; Gehlen et al., 2020; Ahn et al., 2022) and hyperactivity in retinal ganglion cells (Margolis and Detwiler, 2011; Stasheff et al., 2011; Telias et al., 2019; Ahn et al., 2022). This aberrant retinal activity develops with the onset of retinal degeneration and is reported for our age group III and beyond, up until 12 months (Stasheff et al., 2011; Biswas et al., 2014). Moreover, murine models of retinitis pigmentosa provided evidence that this aberrant activity is not confined to the retina but also translated to higher visual areas downstream of the retina, such as the superior colliculus and V1 (Dräger and Hubel, 1978; Ivanova et al., 2016; Wang et al., 2016; Rüland, 2023).

Since mouse models of retinitis pigmentosa are only an abstraction that do not fully encompass the disease progression in humans, the results from these models need to be seen with this caveat. In relation to humans, *rd10* mice show an earlier loss of rod and cone photoreceptors and a critical period that coincides with retinal degeneration. In humans, for most cases, retinal degeneration begins after the critical period (Hamel, 2006; Hartong et al., 2006; Sahel et al., 2014), while there is evidence that altered and reduced visual experience around early childhood can impact visual properties later in life, even if visual experience is restored (Maurer, 2017; Segalowitz et al., 2017; Caravaca-Rodriguez et al., 2022; May et al., 2022; Xiang et al., 2023). This needs to be considered for the assessment of possible vision restoration approaches in the *rd10* mouse model as well as in human patients. The prerequisite for a vision restoration attempt is also a better understanding of the electrophysiological properties of the visual cortex and higher visual areas in regard to aberrant activity from the retina.

Similar to our observations after the onset of retinal degeneration, different murine models of retinitis pigmentosa showed heightened excitability in V1 (Wang et al., 2016; Chen et al., 2020; Leinonen et al., 2022), accompanied by impairments in neurophysiological properties such as visual stimulus direction selectivity (Chen et al., 2016; Leinonen et al., 2022). In addition, human retinitis pigmentosa patients report visual impairments beyond loss of vision, which might be attributed to aberrant retinal activity (Stasheff, 2018). Even though retinal aberrant activity is intensively studied, it is not fully known if this aberrant activity, together with the overall change in input due to retinal degeneration, affects the properties of neurons in V1. This has potential implications for vision restoration methods, since aberrant retinal activity could maintain constant input to V1 (Dräger and Hubel, 1978; Ivanova et al., 2016; Wang et al., 2016; Rüland, 2023) and therefore prevent plastic changes or shifts in the electrophysiological properties of V1 neurons.

## 5. Conclusion

Successful vision restoration therapy after retinal degeneration in retinitis pigmentosa requires intact structures downstream of the retina in the thalamus and visual cortex. Long-term visual deprivation can lead to potential changes in cortical circuits, posing significant constraints on the success of retinal or cortical implants for restoring vision (Caravaca-Rodriguez et al., 2022). Our study provides direct evidence that the electrophysiological properties of V1 neurons remain intact in the *rd10* mouse model after progressing retinal degeneration. We showed distinct changes in the excitability of *rd10* L2/3 PNs after retinal degeneration onset and during the critical period. These changes were, however, transient and V1 neurons appeared similar to control at adult age. In regard to vision restoration therapy, this outcome would be beneficial, as targets downstream of the retina seem to be unaffected by the retinal pathology and still offer potential for electrical or optogenetic stimulation. Improved retinal implants, taking into account ongoing oscillation states of retinal ganglion cells, will offer more efficient stimulation at the level of the retina (Rincón Montes et al., 2019). In addition to retinal stimulation, cortical electrodes with improved long-term stability for chronic recordings and stimulation (Srikantharajah et al., 2021) also provide a promising approach for evoking visual percepts by electrical stimulation of the visual cortex (Beauchamp et al., 2020; Roelfsema, 2020; Fernández et al., 2021). Our results shown here imply that the visual cortex will still be functional and accessible for vision restoration even after prolonged retinal degeneration and blindness.

## Data availability statement

The raw data supporting the conclusions of this article will be made available by the authors, without undue reservation.

## Ethics statement

The animal study was approved by Landesamt für Natur, Umwelt und Verbraucherschutz Nordrhein-Westfalen. The study was conducted in accordance with the local legislation and institutional requirements.

## Author contributions

BK: Conceptualization, Funding acquisition, Project administration, Resources, Supervision, Writing—original draft, Writing—review and editing. CH: Conceptualization, Data curation, Formal analysis, Investigation, Methodology, Software, Visualization, Writing—original draft, Writing—review and editing. TR: Data curation, Software, Writing—review and editing. FM: Conceptualization, Resources, Writing—review and editing. KJ: Writing—review and editing.
