# Supplementary material for: Electrophysiological properties of layer 2/3 pyramidal neurons in the primary visual cortex of a retinitis pigmentosa mouse model (rd10)
Source: Front Cell Neurosci. 2023 Sep 15;17:1258773. doi: 10.3389/fncel.2023.1258773 (PMC10540630; doi:10.3389/fncel.2023.1258773)
Supplement: Supplementary file 1 [file Data_Sheet_1.PDF]

Electrophysiological properties of layer 2/3 pyramidal neurons in the primary visual cortex of a retinitis pigmentosa mouse model (*rd10*)

Claas Halfmann, Thomas Rüland, Frank Müller, Kevin Jehasse, Björn M. Kampa

Supplementary Material

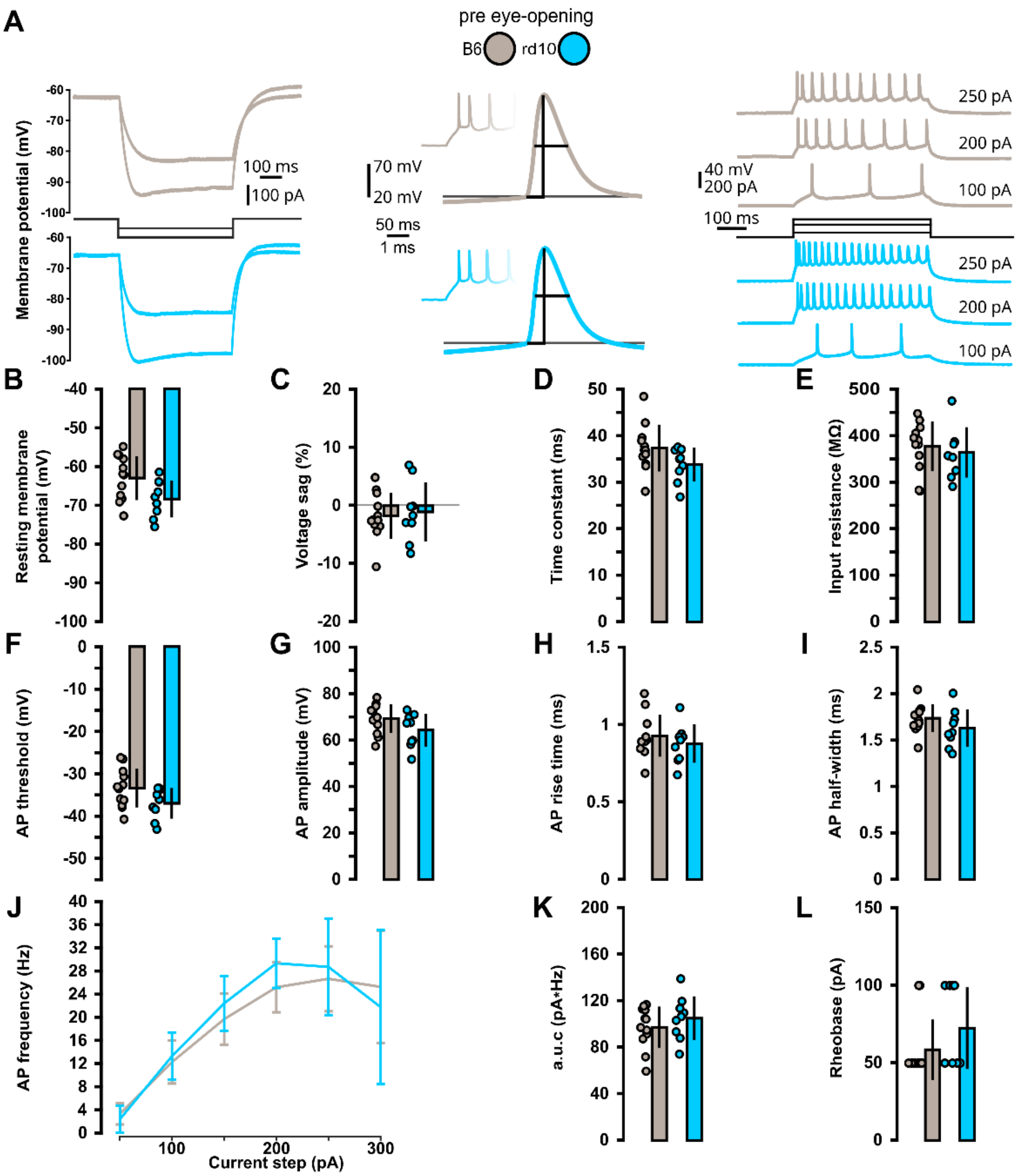

Supplementary Figure 1. Sub- and suprathreshold electrophysiological properties of B6 and *rd10* before eye-opening: **(A)** Exemplary traces of B6 (light gray) and *rd10* (light blue) cells. The first panel part (from left to right) shows examples of voltage responses to hyperpolarizing current steps. Bottom traces show injected current steps (-50 pA and -100 pA). Traces in the background, of the second panel part, show first few action potentials elicited by current step injections. Traces in front are examples of the first action potential of these trains. Vertical and horizontal lines indicate action potential amplitude and halfwidth respectively, bottom line indicates action potential threshold. The third panel shows example traces of action potential firing. For each of the two genotypes one exemplary cell is presented. Each of these cells is shown at three different depolarizing current steps, from rheobase to penultimate current step used. Data presented in bar plots (B-L) is not significant different between B6 and *rd10* (Wilcoxon rank sum test). **(B)** Resting membrane potential **(C)** Voltage sag **(D)** Time constant **(E)** Input resistance **(F)** Action potential threshold **(G)** Action potential amplitude **(H)** Action potential rise time **(I)** Action potential half-width **(J)** FI curve **(K)** area under curve of FI curve **(L)** Rheobase. The complete mean ± SD values according to the bar plots in Supplementary Figure 1B-L are presented in Supplementary Table 11.

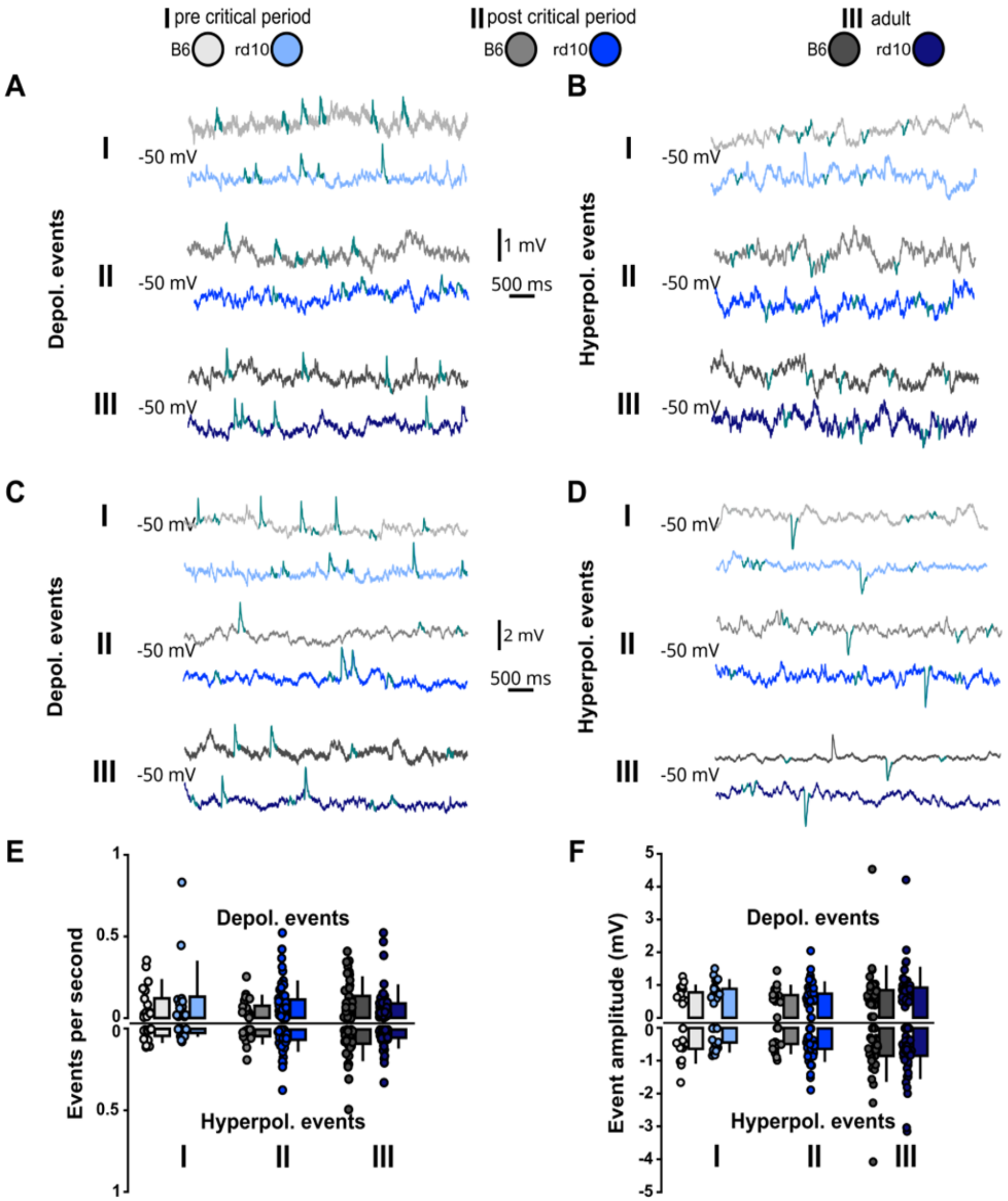

Supplementary Figure 2. Spontaneous subthreshold activity, recorded in current clamp in layer 2/3, additional depolarizing and hyperpolarizing events. **(A-D)** Exemplary traces of spontaneous depolarizing and hyperpolarizing events at membrane potential raised to -50 mV, panel A is the same as article Figure 5A. Supplementary Figure 2, A and B show exemplary **(A)** depolarizing and **(B)** hyperpolarizing events with lower event amplitudes, Panel C and D show exemplary **(C)** depolarizing and **(D)** hyperpolarizing events with higher event amplitudes. Split of exemplary events in depolarizing panel and hyperpolarizing panel allowed better visualization of each event type. **(E)** Mean events per second of depolarizing and hyperpolarizing events, measured at -50 mV membrane potential. Same panel as in article Figure 5B. **(F)** Mean median amplitudes of depolarizing and hyperpolarizing events, measured at -50 mV membrane potential. E-F are the same panels as in article Figure 5C, no additional data, panels E-F were added to complement exemplary traces with the respective data part of the article.

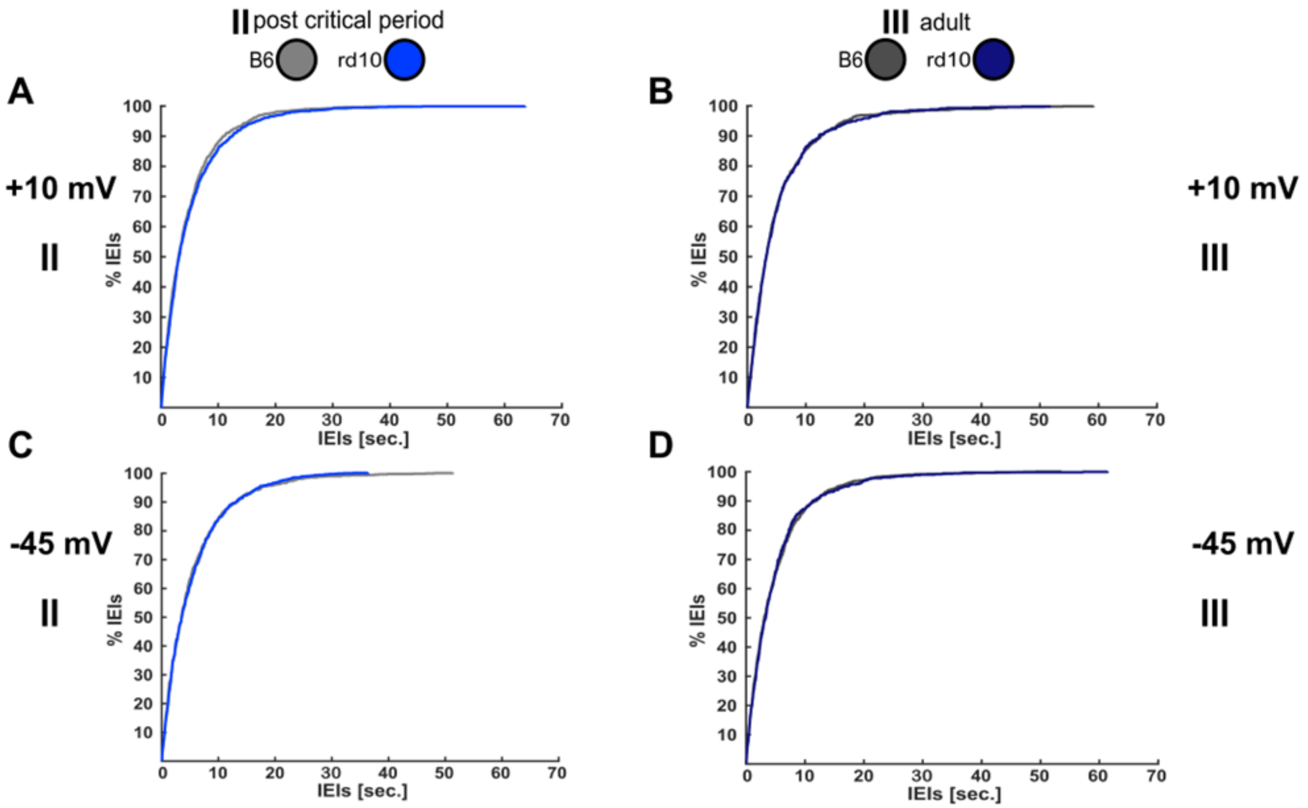

Supplementary Figure 3. Empirical cumulative distributions of interevent intervals (IEIs) of spontaneous activity measurements in voltage clamp. **(A and B)** Interevent intervals of putative inhibitory spontaneous currents (IPSCs +10 mV). **(C and D)** Interevent intervals of putative excitatory spontaneous currents (EPSCs -45 mV). Empirical cumulative distributions did not show significant difference between B6 and *rd10* for age group II **(A and C)** and age group III **(B and D)**, two-sample Kolmogorov-Smirnov test.

1.1 Supplementary Tables

Supplementary Table 1-15, on the following pages, present the values (mean±SD) according to the bar and line graphs in Figure 1-6 and Supplementary Figure 1, in addition these tables present the sample size of mice and cells recorded for each value as well as the statistical tests used and the resulting p-value. Statistical significant values are highlighted by bold print.

Supplementary Table 1: Supra- and Subthreshold membrane properties (Figure 1 and 2)

	**Age Group I**			**Age Group II**			**Age Group III**		
	**B6** Mean±SD (n = number of cells, M = number of mice)	***rd10*** Mean±SD (n = number of cells, M = number of mice)	**Statistics** Test, (p-value)	**B6** Mean±SD (n = number of cells, M = number of mice)	***rd10*** Mean±SD (n = number of cells, M = number of mice)	**Statistics** Test, (p-value)	**B6** Mean±SD (n = number of cells, M = number of mice)	***rd10*** Mean±SD (n = number of cells, M = number of mice)	**Statistics** Test, (p-value)
**Resting membrane potential [mV]**	-71.02±5.96 (n=12) (M=3)	-72.66±5.34 (n=15) (M=4)	WRS, NS (0.510)	-79.33±5.53 (n=35) (M=20)	-78.37±5.72 (n=65) (M=25)	WRS, NS (0.385)	-79.39±4.66 (n=60) (M=28)	-79.85±5.43 (n=66) (M=29)	WRS, NS (0.609)
**Voltage sag [%]**	0.39±3.51 (n=12) (M=3)	0.16±3.29 (n=15) (M=4)	WRS, NS (0.751)	-0.84±3.17 (n=35) (M=20)	0.55±3.33 (n=65) (M=25)	WRS, NS (0.102)	0.75±4.36 (n=60) (M=28)	0.73±3.34 (n=66) (M=29)	WRS, NS (0.912)
**Time constant [ms]**	22.12±6.05 (n=12) (M=3)	18.34±2.87 (n=15) (M=4)	WRS, NS (0.102)	9.23±2.19 (n=35) (M=20)	10.07±2.43 (n=65) (M=25)	WRS, NS (0.070)	8.28±2.23 (n=60) (M=28)	8.67±2.33 (n=66) (M=29)	WRS, NS (0.299)
**Input resistance [MΩ]**	202.37±52.26 (n=12) (M=3)	148.74±28.33 (n=15) (M=4)	**WRS, ** (0.005)**	64.35±18.31 (n=35) (M=20)	78.79±21.38 (n=65) (M=25)	**WRS, *** (0.0003)**	65.74±15.60 (n=60) (M=28)	69.33±19.82 (n=66) (M=29)	WRS, NS (0.264)
**Action potential threshold [mV]**	-38.26±5.04 (n=12) (M=3)	-35.92±3.91 (n=15) (M=4)	WRS, NS (0.113)	-38.60±4.77 (n=35) (M=20)	-36.87±5.03 (n=65) (M=25)	WRS, NS (0.096)	-38.26±4.34 (n=60) (M=28)	-38.73±4.23 (n=66) (M=29)	WRS, NS (0.723)
**Action potential amplitude [mV]**	75.98±6.79 (n=12) (M=3)	76.37±8.38 (n=15) (M=4)	WRS, NS (0.864)	76.09±7.25 (n=35) (M=20)	77.46±7.52 (n=65) (M=25)	WRS, NS (0.233)	76.52±6.26 (n=60) (M=28)	78.32±5.48 (n=66) (M=29)	WRS, NS (0.051)
**Action potential rise time [ms]**	0.75±0.10 (n=12) (M=3)	0.71±0.13 (n=15) (M=4)	WRS, NS (0.420)	0.61±0.06 (n=35) (M=20)	0.67±0.09 (n=65) (M=25)	**WRS, * (0.012)**	0.62±0.08 (n=60) (M=28)	0.61±0.07 (n=66) (M=29)	WRS, NS (0.342)
**Action potential half width [ms]**	1.40±0.19 (n=12) (M=3)	1.33±0.22 (n=15) (M=4)	WRS, NS (0.187)	1.03±0.12 (n=35) (M=20)	1.15±0.17 (n=65) (M=25)	**WRS, *** (0.00035)**	0.99±0.14 (n=60) (M=28)	1.01±0.12 (n=66) (M=29)	WRS, NS (0.522)

Data = mean±SD, n = number of cells, M = number of mice, WRS = Wilcoxon rank sum test, NS = no significant difference, asterisks denote p-values as: * $p < 0.05$, ** $p < 0.01$, *** $p < 0.001$

Supplementary Table 2: Excitability properties I – FI curve (Figure 3B)

	**Age Group I**		**Age Group II**		**Age Group III**	
	**B6** Mean±SD (n = number of cells, M = number of mice)	***rd10*** Mean±SD (n = number of cells, M = number of mice)	**B6** Mean±SD (n = number of cells, M = number of mice)	***rd10*** Mean±SD (n = number of cell, M = number of mice)	**B6** Mean±SD (n = number of cells, M = number of mice)	***rd10*** Mean±SD (n = number of cells, M = number of mice)
**Action potential frequency [Hz] 50 pa**	0±0 (n=12) (M=3)	0±0 (n=15) (M=4)	0±0 (n=35) (M=20)	0±0 (n=65) (M=25)	0±0 (n=60) (M=28)	0±0 (n=66) (M=29)
**Action potential frequency [Hz] 100 pa**	2.35±3.13 (n=12) (M=3)	0.19±0.67 (n=15) (M=4)	0±0 (n=35) (M=20)	0±0 (n=65) (M=25)	0±0 (n=60) (M=28)	0.003 ±0.02 (n=66) (M=29)
**Action potential frequency [Hz] 150 pa**	7.33±5.48 (n=12) (M=3)	3.64±3.66 (n=15) (M=4)	0±0 (n=35) (M=20)	0.5±1.69 (n=65) (M=25)	0.006±0.05 (n=60) (M=28)	0.36±2.09 (n=66) (M=29)
**Action potential frequency [Hz] 200 pa**	13.08±6.21 (n=12) (M=3)	9.43±4.70 (n=15) (M=4)	0.74±2.08 (n=35) (M=20)	2.82±4.18 (n=65) (M=25)	0.48±1.41 (n=60) (M=28)	2.06±4.55 (n=66) (M=29)
**Action potential frequency [Hz] 250 pa**	17.82±6.77 (n=12) (M=3)	15.03±4.64 (n=15) (M=4)	2.25±4.78 (n=35) (M=20)	6.87±6.59 (n=65) (M=25)	2.74±4.09 (n=60) (M=28)	5.26±7.04 (n=66) (M=29)
**Action potential frequency [Hz] 300 pa**	21.92±7.15 (n=12) (M=3)	19.65±4.65 (n=15) (M=4)	5.65±6.68 (n=35) (M=20)	11.26±8.02 (n=65) (M=25)	7.38±6.44 (n=60) (M=28)	9.40±8.56 (n=66) (M=29)
**Action potential frequency [Hz] 350 pa**			10.33±8.20 (n=35) (M=20)	15.72±8.43 (n=65) (M=25)	13.29±7.20 (n=60) (M=28)	14.37±8.92 (n=66) (M=29)
**Action potential frequency [Hz] 400 pa**			15.32±8.73 (n=35) (M=20)	19.91±8.33 (n=65) (M=25)	18.60±7.29 (n=60) (M=28)	18.99±8.79 (n=66) (M=29)
**Action potential frequency [Hz] 450 pa**			19.67±8.61 (n=35) (M=20)	23.45±8.27 (n=65) (M=25)	22.97±7.43 (n=60) (M=28)	23.01±8.84 (n=66) (M=29)
**Action potential frequency [Hz] 500 pa**			23.33±8.54 (n=35) (M=20)	26.56±8.24 (n=65) (M=25)	26.49±7.81 (n=60) (M=28)	26.42±9.05 (n=66) (M=29)

Data = mean±SD, n = number of cells, M = number of mice, Statistical significance was tested with area under curve values, see supplementary table 5.

Supplementary Table 3: Excitability properties II – Action potential amount in stimulation time bin (Figure 3D)

	**Age Group I (250 pA)**			**Age Group II (400 pA)**			**Age Group III (400 pA)**		
	**B6** Mean±SD (n = number of cells, M = number of mice)	***rd10*** Mean±SD (n = number of cells, M = number of mice)	**Statistics** Test, (p-value)	**B6** Mean±SD (n = number of cells, M = number of mice)	***rd10*** Mean±SD (n = number of cells, M = number of mice)	**Statistics** Test, (p-value)	**B6** Mean±SD (n = number of cells, M = number of mice)	***rd10*** Mean±SD (n = number of cells, M = number of mice)	**Statistics** Test, (p-value)
**Action potential amount in time bin 1 0-100 ms**	2.70±0.89 (n=12) (M=3)	2.06±0.69 (n=15) (M=4)	WRS, NS (0.100)	2.16±1.29 (n=35) (M=20)	2.98±1.26 (n=65) (M=25)	**WRS, ** (0.002)**	2.77±1.13 (n=60) (M=28)	2.88±1.43 (n=66) (M=29)	WRS, NS (0.723)
**Action potential amount in time bin 2 100-200 ms**	1.90±0.83 (n=12) (M=3)	1.53±0.48 (n=15) (M=4)	WRS, NS (0.492)	1.57±0.83 (n=35) (M=20)	1.98±0.80 (n=65) (M=25)	**WRS, * (0.014)**	1.89±0.72 (n=60) (M=28)	1.92±0.86 (n=66) (M=29)	WRS, NS (0.994)
**Action potential amount in time bin 3 200-300 ms**	1.48±0.63 (n=12) (M=3)	1.35±0.45 (n=15) (M=4)	WRS, NS (0.844)	1.39±0.83 (n=35) (M=20)	1.78±0.75 (n=65) (M=25)	**WRS, * (0.016)**	1.62±0.63 (n=60) (M=28)	1.69±0.77 (n=66) (M=29)	WRS, NS (0.685)
**Action potential amount in time bin 4 300-400 ms**	1.48±0.55 (n=12) (M=3)	1.28±0.39 (n=15) (M=4)	WRS, NS (0.493)	1.29±0.76 (n=35) (M=20)	1.64±0.74 (n=65) (M=25)	**WRS, * (0.015)**	1.56±0.65 (n=60) (M=28)	1.52±0.73 (n=66) (M=29)	WRS, NS (0.807)
**Action potential amount in time bin 5 400-500 ms**	1.34±0.59 (n=12) (M=3)	1.28±0.47 (n=15) (M=4)	WRS, NS (0.825)	1.24±yy (n=35) (M=20)	1.57±0.73 (n=65) (M=25)	**WRS, * (0.021)**	1.45±0.66 (n=60) (M=28)	1.46±0.72 (n=66) (M=29)	WRS, NS (0.938)

Data = mean±SD, n = number of cells, M = number of mice, WRS = Wilcoxon rank sum test, NS = no significant difference, asterisks denote p-values as: * p < 0.05, ** p < 0.01, *** p < 0.001

Supplementary Table 4: Excitability properties III – Adaptation ratio (Figure 3E)

	**Age Group I**			**Age Group II**			**Age Group III**		
	**B6** Mean±SD (n = number of cells, M = number of mice)	***rd10*** Mean±SD (n = number of cells, M = number of mice)	**Statistics** Test, (p-value)	**B6** Mean±SD (n = number of cells, M = number of mice)	***rd10*** Mean±SD (n = number of cells, M = number of mice)	**Statistics** Test, (p-value)	**B6** Mean±SD (n = number of cells, M = number of mice)	***rd10*** Mean±SD (n = number of cells, M = number of mice)	**Statistics** Test, (p-value)
**Adaptation ratio [ISI-1 / ISI-9]**	0.31±0.06 (n=11) (M=3)	0.35±0.06 (n=15) (M=4)	WRS, NS (0.299)	0.29±0.08 (n=22) (M=9)	0.28±0.07 (n=48) (M=14)	WRS, NS (0.690)	0.24±0.07 (n=47) (M=20)	0.27±0.06 (n=54) (M=19)	WRS, NS (0.082)
**Adaptation ratio [ISI-2 / ISI-9]**	0.53±0.09 (n=11) (M=3)	0.61±0.07 (n=15) (M=4)	WRS, NS (0.062)	0.61±0.10 (n=22) (M=9)	0.58±0.09 (n=48) (M=14)	WRS, NS (0.222)	0.58±0.10 (n=47) (M=20)	0.55±0.09 (n=54) (M=19)	WRS, NS (0.050)
**Adaptation ratio [ISI-3 / ISI-9]**	0.66±0.12 (n=11) (M=3)	0.73±0.07 (n=15) (M=4)	WRS, NS (0.161)	0.72±0.08 (n=22) (M=9)	0.72±0.09 (n=48) (M=14)	WRS, NS (0.681)	0.72±0.10 (n=47) (M=20)	0.70±0.08 (n=54) (M=19)	WRS, NS (0.071)
**Adaptation ratio [ISI-4 / ISI-9]**	0.76±0.13 (n=11) (M=3)	0.81±0.06 (n=15) (M=4)	WRS, NS (0.604)	0.79±0.08 (n=22) (M=9)	0.80±0.08 (n=48) (M=14)	WRS, NS (0.573)	0.80±0.08 (n=47) (M=20)	0.79±0.07 (n=54) (M=19)	WRS, NS (0.135)
**Adaptation ratio [ISI-5 / ISI-9]**	0.81±0.13 (n=11) (M=3)	0.86±0.05 (n=15) (M=4)	WRS, NS (0.604)	0.84±0.07 (n=22) (M=9)	0.86±0.07 (n=48) (M=14)	WRS, NS (0.296)	0.86±0.07 (n=47) (M=20)	0.85±0.06 (n=54) (M=19)	WRS, NS (0.432)
**Adaptation ratio [ISI-6 / ISI-9]**	0.87±0.09 (n=11) (M=3)	0.91±0.04 (n=15) (M=4)	WRS, NS (0.499)	0.89±0.05 (n=22) (M=9)	0.90±0.05 (n=48) (M=14)	WRS, NS (0.186)	0.91±0.06 (n=47) (M=20)	0.89±0.04 (n=54) (M=19)	WRS, NS (0.439)
**Adaptation ratio [ISI-7 / ISI-9]**	0.94±0.08 (n=11) (M=3)	0.93±0.04 (n=15) (M=4)	WRS, NS (0.568)	0.93±0.05 (n=22) (M=9)	0.94±0.04 (n=48) (M=14)	WRS, NS (0.075)	0.94±0.04 (n=47) (M=20)	0.93±0.04 (n=54) (M=19)	WRS, NS (0.148)
**Adaptation ratio [ISI-8 / ISI-9]**	1.02±0.15 (n=11) (M=3)	0.96±0.03 (n=15) (M=4)	WRS, NS (0.406)	0.97±0.03 (n=22) (M=9)	0.97±0.04 (n=48) (M=14)	WRS, NS (0.104)	0.98±0.03 (n=47) (M=20)	0.97±0.02 (n=54) (M=19)	WRS, NS (0.309)

Data = mean±SD, n = number of cells, M = number of mice, WRS = Wilcoxon rank sum test, NS = no significant difference, asterisks denote p-values as: * $p < 0.05$, ** $p < 0.01$, *** $p < 0.001$

Supplementary Table 5: Excitability properties IV – Area under curve (a.u.c), Rheobase, Spontaneous activity-Action potentials per second (Figure 3B [inset], C and G)

	**Age Group I**			**Age Group II**			**Age Group III**		
	**B6** Mean±SD (n = number of cells, M = number of mice)	***rd10*** Mean±SD (n = number of cells, M = number of mice)	**Statistics** Test, (p-value)	**B6** Mean±SD (n = number of cells, M = number of mice)	***rd10*** Mean±SD (n = number of cells, M = number of mice)	**Statistics** Test, (p-value)	**B6** Mean±SD (n = number of cells, M = number of mice)	***rd10*** Mean±SD (n = number of cells, M = number of mice)	**Statistics** Test, (p-value)
**Area under curve [pA*Hz]**	49.73±23.65 (n=12) (M=3)	36.52±14.63 (n=15) (M=4)	WRS, NS (0.232)	64.31±41.12 (n=35) (M=20)	92.22±46.72 (n=65) (M=25)	**WRS, ** (0.0026)**	77.33±34.71 (n=60) (M=28)	85.04±50.06 (n=66) (M=29)	WRS, NS (0.655)
**Rheobase [pA]**	121.66±40.19 (n=12) (M=3)	154±40.49 (n=15) (M=4)	WRS, NS (0.073)	314.28±65.49 (n=35) (M=20)	246.46±61.09 (n=65) (M=25)	**WRS, *** (0.0000041)**	293±60.62 (n=60) (M=28)	267.27±71.39 (n=66) (M=29)	WRS, NS (0.057)
**Spontaneous activity action potentials at -40 mV [Action potentials per second]**	1.79±1.86 (n=8) (M=3)	1.78±1.67 (n=12) (M=4)	WRS, NS (0.847)	0.81±1.14 (n=12) (M=5)	0.60±0.58 (n=23) (M=12)	WRS, NS (0.821)	0.49±0.99 (n=14) (M=7)	0.85±1.38 (n=33) (M=12)	WRS, NS (0.053)

Data = mean±SD, n = number of cells, M = number of mice, WRS = Wilcoxon rank sum test, NS = no significant difference, asterisks denote p-values as: * p < 0.05, ** p < 0.01, *** p < 0.001

Supplementary Table 6: Extracellular stimulation in layer 2/3, recording in current clamp in layer 2/3 – paired pulse ratio (Figure 4C-D)

	**Age Group I**			**Age Group II**			**Age Group III**		
	**B6** Mean±SD (n = number of cells, M = number of mice)	***rd10*** Mean±SD (n = number of cells, M = number of mice)	**Statistics** Test, (p-value)	**B6** Mean±SD (n = number of cells, M = number of mice)	***rd10*** Mean±SD (n = number of cells, M = number of mice)	**Statistics** Test, (p-value)	**B6** Mean±SD (n = number of cells, M = number of mice)	***rd10*** Mean±SD (n = number of cells, M = number of mice)	**Statistics** Test, (p-value)
**Paired pulse ratio 20 Hz PPR (2Pulse /1 Pulse)**	0.73±0.10 (n=8) (M=2)	0.78±0.12 (n=13) (M=4)	WRS, NS (0.262)	0.96±0.15 (n=13) (M=6)	0.97±0.09 (n=16) (M=8)	WRS, NS (0.878)	0.99±0.09 (n=21) (M=9)	0.97±0.08 (n=29) (M=13)	WRS, NS (0.183)
**Paired pulse ratio 20 Hz PPR (3Pulse /1 Pulse)**	0.63±0.15 (n=8) (M=2)	0.66±0.12 (n=13) (M=4)	WRS, NS (0.491)	0.93±0.15 (n=13) (M=6)	0.91±0.12 (n=16) (M=8)	WRS, NS (0.392)	0.94±0.12 (n=21) (M=9)	0.91±0.09 (n=29) (M=13)	WRS, NS (0.255)
**Paired pulse ratio 20 Hz PPR (4Pulse /1 Pulse)**	0.55±0.11 (n=8) (M=2)	0.61±0.11 (n=13) (M=4)	WRS, NS (0.328)	0.89±0.16 (n=13) (M=6)	0.87±0.13 (n=16) (M=8)	WRS, NS (0.584)	0.89±0.11 (n=21) (M=9)	0.87±0.12 (n=29) (M=13)	WRS, NS (0.306)
**Paired pulse ratio 20 Hz PPR (5Pulse /1 Pulse)**	0.48±0.09 (n=8) (M=2)	0.55±0.09 (n=13) (M=4)	WRS, NS (0.158)	0.87±0.16 (n=13) (M=6)	0.83±0.13 (n=16) (M=8)	WRS, NS (0.167)	0.86±0.14 (n=21) (M=9)	0.84±0.12 (n=29) (M=13)	WRS, NS (0.297)
**Paired pulse ratio 40 Hz PPR (2Pulse /1 Pulse)**	0.96±0.32 (n=8) (M=2)	0.89±0.19 (n=13) (M=4)	WRS, NS (0.405)	1.33±0.15 (n=14) (M=6)	1.10±0.18 (n=19) (M=9)	**WRS, *** (0.0007)**	1.12±0.23 (n=24) (M=10)	1.17±0.17 (n=37) (M=14)	WRS, NS (0.447)

Data = mean±SD, n = number of cells, M = number of mice, WRS = Wilcoxon rank sum test, NS = no significant difference, asterisks denote p-values as: * p < 0.05, ** p < 0.01, *** p < 0.001, please note: age group I B6 data (20 Hz) is failure corrected, see material and method section.

Supplementary Table 7: Spontaneous subthreshold activity, recordings in current clamp in layer 2/3 (Figure 5A-C)

	**Age Group I**			**Age Group II**			**Age Group III**		
	**B6** Mean±SD (n = number of cells, M = number of mice)	***rd10*** Mean±SD (n = number of cells, M = number of mice)	**Statistics** Test, (p-value)	**B6** Mean±SD (n = number of cells, M = number of mice)	***rd10*** Mean±SD (n = number of cells, M = number of mice)	**Statistics** Test, (p-value)	**B6** Mean±SD (n = number of cells, M = number of mice)	***rd10*** Mean±SD (n = number of cells, M = number of mice)	**Statistics** Test, (p-value)
**Depol. events at -50 mV Events per second**	0.12±0.12 (n=13) (M=3)	0.13±0.22 (n=15) (M=4)	WRS, NS (0.534)	0.07±0.07 (n=14) (M=5)	0.11±0.12 (n=44) (M=15)	WRS, NS (0.383)	0.13±0.12 (n=31) (M=14)	0.09±0.12 (n=41) (M=16)	WRS, NS (0.075)
**Depol. events at -50 mV Amplitude [mV]**	0.77±0.24 (n=13) (M=3)	0.88±0.29 (n=15) (M=4)	WRS, NS (0.381)	0.69±0.32 (n=14) (M=5)	0.73±0.38 (n=44) (M=15)	WRS, NS (0.744)	0.84±0.76 (n=31) (M=14)	0.91±0.64 (n=41) (M=16)	WRS, NS (0.433)
**Hyperpol. events at -50 mV Events per second**	0.04±0.04 (n=13) (M=3)	0.02±0.02 (n=15) (M=4)	WRS, NS (0.516)	0.04±0.05 (n=14) (M=5)	0.06±0.07 (n=44) (M=15)	WRS, NS (0.207)	0.09±0.11 (n=31) (M=14)	0.05±0.06 (n=41) (M=16)	WRS, NS (0.078)
**Hyperpol. events at -50 mV Amplitude [mV]**	0.64±0.45 (n=13) (M=3)	0.44±0.31 (n=15) (M=4)	WRS, NS (0.405)	0.49±0.31 (n=14) (M=5)	0.63±0.39 (n=44) (M=15)	WRS, NS (0.353)	0.84±0.79 (n=31) (M=14)	0.84±0.71 (n=41) (M=16)	WRS, NS (0.802)

Data = mean±SD, n = number of cells, M = number of mice, WRS = Wilcoxon rank sum test, NS = no significant difference, asterisks denote p-values as: * $p < 0.05$, ** $p < 0.01$, *** $p < 0.001$, Amplitude is mean median amplitude

Supplementary Table 8: Spontaneous subthreshold activity, recordings in voltage clamp in layer 2/3 (Figure 5E-F)

	**Age Group II**			**Age Group III**		
	**B6** Mean±SD (n = number of cells, M = number of mice)	***rd10*** Mean±SD (n = number of cells, M = number of mice)	**Statistics** Test, (p-value)	**B6** Mean±SD (n = number of cells, M = number of mice)	***rd10*** Mean±SD (n = number of cells, M = number of mice)	**Statistics** Test, (p-value)
**Putative inhibitory events at +10 mV Events per second**	0.19±0.05 (n=16) (M=11)	0.17±0.06 (n=19) (M=8)	WRS, NS (0.345)	0.17±0.05 (n=18) (M=11)	0.18±0.05 (n=11) (M=7)	WRS, NS (0.653)
**Putative inhibitory events at +10 mV Amplitude [pA]**	16.60±15.82 (n=16) (M=11)	13.07±8.12 (n=19) (M=8)	WRS, NS (0.380)	10.05±4.09 (n=18) (M=11)	17.49±6.15 (n=11) (M=7)	**WRS, ** (0.0018)**
**Putative excitatory events at -45 mV Events per second**	0.16±0.07 (n=18) (M=13)	0.17±0.04 (n=24) (M=11)	WRS, NS (0.380)	0.19±0.04 (n=19) (M=12)	0.18±0.08 (n=17) (M=9)	WRS, NS (0.634)
**Putative excitatory events at -45 mV Amplitude [pA]**	7.99±1.99 (n=18) (M=13)	9.15±3.14 (n=24) (M=11)	WRS, NS (0.303)	8.06±2.97 (n=19) (M=12)	9.17±3.56 (n=17) (M=9)	WRS, NS (0.657)

Data = mean±SD, n = number of cells, M = number of mice, WRS = Wilcoxon rank sum test, NS = no significant difference, asterisks denote p-values as: * $p < 0.05$, ** $p < 0.01$, *** $p < 0.001$, Amplitude is mean median amplitude

Supplementary Table 9: Extracellular stimulation in layer 4, recording in voltage clamp in layer 2/3 at +10 mV (putative inhibitory events) – paired pulse ratio (Figure 6C-D)

	Age Group II			Age Group III		
	**B6**<br>Mean±SD<br>(n = number of cells,<br>M = number of mice)	***rd10***<br>Mean±SD<br>(n = number of cells,<br>M = number of mice)	**Statistics**<br>Test,<br>(p-value)	**B6**<br>Mean±SD<br>(n = number of cells,<br>M = number of mice)	***rd10***<br>Mean±SD<br>(n = number of cells,<br>M = number of mice)	**Statistics**<br>Test,<br>(p-value)
**Paired pulse ratio 20 Hz PPR (2Pulse /1 Pulse)**	0.71±0.21 (n=10) (M=7)	0.68±0.22 (n=13) (M=7)	WRS, NS (0.687)	0.78±0.14 (n=12) (M=7)	0.68±0.21 (n=8) (M=5)	WRS, NS (0.563)
**Paired pulse ratio 20 Hz PPR (3Pulse /1 Pulse)**	0.68±0.23 (n=10) (M=7)	0.58±0.18 (n=13) (M=7)	WRS, NS (0.402)	0.69±0.20 (n=12) (M=7)	0.63±0.22 (n=8) (M=5)	WRS, NS (0.616)
**Paired pulse ratio 20 Hz PPR (4Pulse /1 Pulse)**	0.69±0.25 (n=10) (M=7)	0.67±0.45 (n=13) (M=7)	WRS, NS (0.368)	0.73±0.22 (n=12) (M=7)	0.58±0.22 (n=8) (M=5)	WRS, NS (0.375)
**Paired pulse ratio 20 Hz PPR (5Pulse /1 Pulse)**	0.62±0.30 (n=10) (M=7)	0.55±0.21 (n=13) (M=7)	WRS, NS (0.556)	0.75±0.30 (n=12) (M=7)	0.62±0.28 (n=8) (M=5)	WRS, NS (0.375)
**Paired pulse ratio 40 Hz PPR (2Pulse /1 Pulse)**	0.69±0.19 (n=11) (M=8)	0.66±0.26 (n=17) (M=8)	WRS, NS (0.572)	0.83±0.31 (n=16) (M=10)	0.85±0.43 (n=10) (M=6)	WRS, NS (1)

Data = mean±SD, n = number of cells, M = number of mice, WRS = Wilcoxon rank sum test, NS = no significant difference, asterisks denote p-values as: * $p < 0.05$, ** $p < 0.01$, *** $p < 0.001$, please note: 20 Hz data is failure corrected, see material and method section.

Supplementary Table 10: Extracellular stimulation in layer 4, recording in voltage clamp in layer 2/3 at -45 mV (putative excitatory events) – paired pulse ratio (Figure 6E-F)

	**Age Group II**			**Age Group III**		
	**B6** Mean±SD (n = number of cells, M = number of mice)	***rd10*** Mean±SD (n = number of cells, M = number of mice)	**Statistics** Test, (p-value)	**B6** Mean±SD (n = number of cells, M = number of mice)	***rd10*** Mean±SD (n = number of cells, M = number of mice)	**Statistics** Test, (p-value)
**Paired pulse ratio 20 Hz PPR (2Pulse /1 Pulse)**	1.05±0.14 (n=17) (M=13)	1.13±0.15 (n=14) (M=7)	WRS, NS (0.065)	1.04±0.08 (n=19) (M=12)	1.05±0.19 (n=7) (M=5)	WRS, NS (1)
**Paired pulse ratio 20 Hz PPR (3Pulse /1 Pulse)**	1.01±0.14 (n=17) (M=13)	1.10±0.16 (n=14) (M=7)	WRS, NS (0.077)	1.02±0.11 (n=19) (M=12)	1.05±0.21 (n=7) (M=5)	WRS, NS (0.817)
**Paired pulse ratio 20 Hz PPR (4Pulse /1 Pulse)**	0.98±0.17 (n=17) (M=13)	1.06±0.16 (n=14) (M=7)	WRS, NS (0.065)	0.99±0.09 (n=19) (M=12)	1.02±0.26 (n=7) (M=5)	WRS, NS (0.817)
**Paired pulse ratio 20 Hz PPR (5Pulse /1 Pulse)**	0.96±0.14 (n=17) (M=13)	0.99±0.14 (n=14) (M=7)	WRS, NS (0.137)	0.96±0.09 (n=19) (M=12)	1.00±0.27 (n=7) (M=5)	WRS, NS (1)
**Paired pulse ratio 40 Hz PPR (2Pulse /1 Pulse)**	1.27±0.38 (n=18) (M=13)	1.34±0.33 (n=20) (M=8)	WRS, NS (0.248)	1.24±0.20 (n=19) (M=12)	1.30±0.24 (n=7) (M=5)	WRS, NS (0.418)

Data = mean±SD, n = number of cells, M = number of mice, WRS = Wilcoxon rank sum test, NS = no significant difference, asterisks denote p-values as: * $p < 0.05$, ** $p < 0.01$, *** $p < 0.001$, please note: 20 Hz data is failure corrected, see material and method section.

Supplementary Table 11: Sub- and suprathreshold membrane properties before eye-opening (Supplementary Figure 1)

	**Pre eye-opening**		
	**B6**<br>Mean±SD<br>(n = number of cells,<br>M = number of mice)	***rd10***<br>Mean±SD<br>(n = number of cells,<br>M = number of mice)	**Statistics**<br>Test,<br>(p-value)
**Resting membrane potential [mV]**	-62.56±5.82<br>(n=12)<br>(M=2)	-68.08±4.86<br>(n=9)<br>(M=2)	WRS, NS<br>(0.051)
**Voltage sag [%]**	-1.76±3.98<br>(n=12)<br>(M=2)	-1.06±5.15<br>(n=9)<br>(M=2)	WRS, NS<br>(0.749)
**Time constant [ms]**	37.11±4.99<br>(n=12)<br>(M=2)	33.61±3.58<br>(n=9)<br>(M=2)	WRS, NS<br>(0.109)
**Input resistance [MΩ]**	376.98±53.42<br>(n=12)<br>(M=2)	364.29±54.07<br>(n=9)<br>(M=2)	WRS, NS<br>(0.414)
**Action potential threshold [mV]**	-33.19±4.53<br>(n=12)<br>(M=2)	-36.76±3.59<br>(n=9)<br>(M=2)	WRS, NS<br>(0.082)
**Action potential amplitude [mV]**	69.27±6.25<br>(n=12)<br>(M=2)	64.28±7.12<br>(n=9)<br>(M=2)	WRS, NS<br>(0.126)
**Action potential rise time [ms]**	0.93±0.13<br>(n=12)<br>(M=2)	0.88±0.12<br>(n=9)<br>(M=2)	WRS, NS<br>(0.669)
**Action potential half width [ms]**	1.73±0.15<br>(n=12)<br>(M=2)	1.63±0.20<br>(n=9)<br>(M=2)	WRS, NS<br>(0.145)
**Area under curve [pA*Hz]**	97.78± 17.93<br>(n=12)<br>(M=2)	105.65± 18.93<br>(n=9)<br>(M=2)	WRS, NS<br>(0.455)
**Rheobase [pA]**	58.33±19.46<br>(n=12)<br>(M=2)	72.22±26.35<br>(n=9)<br>(M=2)	WRS, NS<br>(0.188)

Data = mean±SD, n = number of cells, M = number of mice, WRS = Wilcoxon rank sum test, NS = no significant difference, asterisks denote p-values as: * $p < 0.05$, ** $p < 0.01$, *** $p < 0.001$

Supplementary Table 12: Overview of inter-age group multiple comparison test of B6 sub- and suprathreshold membrane properties, post hoc Bonferroni corrected, following a Kruskal Wallis test

	AG-0	AG-I	AG-II	AG-III
**Resting membrane potential [mV]**	Sig. diff. to AG-II (***) and AG-III (***), NS to AG-I	Sig. diff. to AG-II (**) and AG-III (**), NS to AG-0	Sig. diff. to AG-0 (***) and AG-I (**), NS to AG-III	Sig. diff. to AG-0 (***) and AG-I (**), NS to AG-II
**Voltage sag [%]**	NS to all other AGs	NS to all other AGs	NS to all other AGs	NS to all other AGs
**Time constant [ms]**	Sig. diff. to AG-II (***) and AG-III (***), NS to AG-I	Sig. diff. to AG-II (***) and AG-III (***), NS to AG-0	Sig. diff. to AG-0 (***),AG-I (***), NS to AG-III	Sig. diff. to AG-0 (***),AG-I (***), NS to AG-II
**Input resistance [MΩ]**	Sig. diff. to AG-II (***) and AG-III (***), NS to AG-I	Sig. diff. to AG-II (***) and AG-III (***), NS to AG-0	Sig. diff. to AG-0 (***) and AG-I (***), NS to AG-III	Sig. diff. to AG-0 (***) and AG-I (***), NS to AG-II
**Action potential threshold [mV]**	Sig. diff. to AG-II (**) and AG-III (**), NS to AG-I	NS to all other AGs	Sig. diff. to AG-0 (**), NS to AG-I and AG-III	Sig. diff. to AG-0 (**), NS to AG-I and AG-II
**Action potential amplitude [mV]**	Sig. diff. to AG-II (*) and AG-III (**), NS to AG-I	NS to all other AGs	Sig. diff. to AG-0 (*), NS to AG-I and AG-III	Sig. diff. to AG-0 (**), NS to AG-I and AG-II
**Action potential rise time [ms]**	Sig. diff. to AG-II (***) and AG-III (***), NS to AG-I	Sig. diff. to AG-II (**) and AG-III (**), NS to AG-0	Sig. diff. to AG-0 (***) and AG-I (**), NS to AG-III	Sig. diff. to AG-0 (***) and AG-I (**), NS to AG-II
**Action potential half width [ms]**	Sig. diff. to AG-II (***) and AG-III (***), NS to AG-I	Sig. diff. to AG-II (***) and AG-III (***), NS to AG-0	Sig. diff. to AG-0 (***) and AG-I (***), NS to AG-III	Sig. diff. to AG-0 (***) and AG-I (***), NS to AG-II

AG-0 = age group pre eye-opening, sig. diff. = significant difference, NS = no significant difference, asterisks denote p-values as: * $p < 0.05$, ** $p < 0.01$, *** $p < 0.001$

Supplementary Table 13: Overview of inter-age group multiple comparison test of B6 rheobase and action potential firing frequency, post hoc Bonferroni corrected, following a Kruskal Wallis test

	**AG-0**	**AG-I**	**AG-II**	**AG-III**
**Rheobase [pA]**	Sig. diff. to AG-II (***) and AG-III (***), NS to AG-I	Sig. diff. to AG-II (***) and AG-III (***), NS to AG-0	Sig. diff. to AG-0 (***) and AG-I (***), NS to AG-III	Sig. diff. to AG-0 (***) and AG-I (***), NS to AG-II
**AP firing frequency at 100 pA [Hz]**	Sig. diff. to AG-I (***), AG-II (***) and AG-III (***)	Sig. diff. to AG-0 (***), AG-II (***) and AG-III (***)	Sig. diff. to AG-0 (***),AG-I (***), NS to AG-III	Sig. diff. to AG-0 (***),AG-I (***), NS to AG-II
**AP firing frequency at 150 pA [Hz]**	Sig. diff. to AG-II (**) and AG-III (***), NS to AG-I	Sig. diff. to AG-III (***), NS to AG-0 and AG-II	Sig. diff. to AG-0 (**) and AG-III (***), NS to AG-I	Sig. diff. to AG-0 (***) and AG-I (***), AG-II (***)
**AP firing frequency at 200 pA [Hz]**	Sig. diff. to AG-II (***) and AG-III (***), NS to AG-I	Sig. diff. to AG-II (**) and AG-III (***), NS to AG-0	Sig. diff. to AG-0 (***) and AG-I (**), AG-III (***)	Sig. diff. to AG-0 (***) and AG-I (***), AG-II (***)
**AP firing frequency at 250 pA [Hz]**	Sig. diff. to AG-II (***) and AG-III (***), NS to AG-I	Sig. diff. to AG-II (***) and AG-III (***), NS to AG-0	Sig. diff. to AG-0 (***),AG-I (***), NS to AG-III	Sig. diff. to AG-0 (***),AG-I (***), NS to AG-II
**AP firing frequency at 300 pA [Hz]**	Sig. diff. to AG-II (***) and AG-III (***), NS to AG-I	Sig. diff. to AG-II (***) and AG-III (***), NS to AG-0	Sig. diff. to AG-0 (***),AG-I (***), NS to AG-III	Sig. diff. to AG-0 (***),AG-I (***), NS to AG-II

Supplementary table 13 contains all AP firing frequencies at pA steps which were shared by all age groups (100 pA to 300 pA). AG-0 = age group pre eye-opening, sig. diff. = significant difference, NS = no significant difference, asterisks denote p-values as: * $p < 0.05$, ** $p < 0.01$, *** $p < 0.001$

Supplementary Table 14: Overview of inter-age group multiple comparison test of *rd10* sub- and suprathreshold membrane properties, post hoc Bonferroni corrected, following a Kruskal Wallis test

	**AG-0**	**AG-I**	**AG-II**	**AG-III**
**Resting membrane potential [mV]**	Sig. diff. to AG-II (***) and AG-III (***), NS to AG-I	Sig. diff. to AG-II (**) and AG-III (***), NS to AG-0	Sig. diff. to AG-0 (***) and AG-I (***), NS to AG-III	Sig. diff. to AG-0 (***) and AG-I (***), NS to AG-II
**Voltage sag [%]**	NS to all other AGs	NS to all other AGs	NS to all other AGs	NS to all other AGs
**Time constant [ms]**	Sig. diff. to AG-II (***) and AG-III (***), NS to AG-I	Sig. diff. to AG-II (***) and AG-III (***), NS to AG-0	Sig. diff. to AG-0 (***),AG-I (***) and AG-III (**)	Sig. diff. to AG-0 (***),AG-I (***) and AG-II (**)
**Input resistance [MΩ]**	Sig. diff. to AG-II (***) and AG-III (***), NS to AG-I	Sig. diff. to AG-II (***) and AG-III (***), NS to AG-0	Sig. diff. to AG-0 (***) and AG-I (***), NS to AG-III	Sig. diff. to AG-0 (***) and AG-I (***), NS to AG-II
**Action potential threshold [mV]**	NS to all other AGs	NS to all other AGs	NS to all other AGs	NS to all other AGs
**Action potential amplitude [mV]**	Sig. diff. to AG-I (*), AG-II (***) and AG-III (***)	Sig. diff. to AG-0 (*), NS to AG-II and AG-III	Sig. diff. to AG-0 (***), NS to AG-I and AG-III	Sig. diff. to AG-0 (***), NS to AG-I and AG-II
**Action potential rise time [ms]**	Sig. diff. to AG-II (**) and AG-III (***), NS to AG-I	Sig. diff. to AG-III (*), NS to AG-0 and AG-II	Sig. diff. to AG-0 (**) and AG-III (**), NS to AG-I	Sig. diff. to AG-0 (***),AG-I (*) and AG-II (**)
**Action potential half width [ms]**	Sig. diff. to AG-II (**) and AG-III (***), NS to AG-I	Sig. diff. to AG-III (***), NS to AG-0 and AG-II	Sig. diff. to AG-0 (**) and AG-III (***), NS to AG-I	Sig. diff. to AG-0 (***),AG-I (***) and AG-II (***)

AG-0 = age group pre eye-opening, sig. diff. = significant difference, NS = no significant difference, asterisks denote p-values as: * $p < 0.05$, ** $p < 0.01$, *** $p < 0.001$

Supplementary Table 15: Overview of inter-age group multiple comparison test of *rd10* rheobase and action potential firing frequency, post hoc Bonferroni corrected, following a Kruskal Wallis test

	**AG-0**	**AG-I**	**AG-II**	**AG-III**
**Rheobase [pA]**	Sig. diff. to AG-II (***) and AG-III (***), NS to AG-I	Sig. diff. to AG-II (***) and AG-III (***), NS to AG-0	Sig. diff. to AG-0 (***) and AG-I (***), NS to AG-III	Sig. diff. to AG-0 (***) and AG-I (***), NS to AG-II
**AP firing frequency at 100 pA [Hz]**	Sig. diff. to AG-I (***), AG-II (***) and AG-III (***)	Sig. diff. to AG-0 (***), NS to AG-II and AG-III	Sig. diff. to AG-0 (***), NS to AG-I and AG-III	Sig. diff. to AG-0 (***), NS to AG-I and AG-II
**AP firing frequency at 150 pA [Hz]**	Sig. diff. to AG-II (**) and AG-III (***), NS to AG-I	Sig. diff. to AG-III (***), NS to AG-0 and AG-II	Sig. diff. to AG-0 (**) and AG-III (***), NS to AG-I	Sig. diff. to AG-0 (***) and AG-I (***), AG-II (***)
**AP firing frequency at 200 pA [Hz]**	Sig. diff. to AG-II (***) and AG-III (***), NS to AG-I	Sig. diff. to AG-II (**) and AG-III (***), NS to AG-0	Sig. diff. to AG-0 (***) and AG-I (**), AG-III (***)	Sig. diff. to AG-0 (***) and AG-I (***), AG-II (***)
**AP firing frequency at 250 pA [Hz]**	Sig. diff. to AG-II (***) and AG-III (***), NS to AG-I	Sig. diff. to AG-II (**) and AG-III (***), NS to AG-0	Sig. diff. to AG-0 (***),AG-I (**), NS to AG-III	Sig. diff. to AG-0 (***),AG-I (***), NS to AG-II
**AP firing frequency at 300 pA [Hz]**	Sig. diff. to AG-III (*), NS to AG-I, AG-II	Sig. diff. to AG-II (**) and AG-III (***), NS to AG-0	Sig. diff. to AG-I (**), NS to AG-0, AG-III	Sig. diff. to AG-0 (*),AG-I (***), NS to AG-II

Supplementary table 15 contains all AP firing frequencies at pA steps which were shared by all age groups (100 pA to 300 pA). AG-0 = age group pre eye-opening, sig. diff. = significant difference, NS = no significant difference, asterisks denote p-values as: * $p < 0.05$, ** $p < 0.01$, *** $p < 0.001$
